# Examining longitudinal disparities in COVID-19 prevalence in the U.S.: a county level growth rate perspective

**DOI:** 10.1080/07853890.2022.2069852

**Published:** 2022-05-06

**Authors:** Amin Kiaghadi, Omolola E. Adepoju, Hanadi S. Rifai, Winston Liaw, Lechauncy D. Woodard

**Affiliations:** a Civil and Environmental Engineering, University of Houston, Houston, TX, USA; b Health Systems and Population Health Sciences, College of Medicine, University of Houston, Houston, TX, USA

**Keywords:** Sociodemographic determinants, stay home order, PM_2.5_

## Abstract

**Background:** The objectives of the present study are to understand the longitudinal variability in COVID-19 reported cases at the county level and to associate the observed rates of infection with the adoption and lifting of stay-home orders.

**Materials and Methods:** The study uses the trajectory of the pandemic in a county and controls for social and economic risk factors, physical environment, and health behaviors to elucidate the social determinants contributing to the observed rates of infection.

**Results and conclusion:** Results indicated that counties with higher percentages of young individuals, racial and ethnic minorities and, higher population densities experienced greater difficulty suppressing transmission.Except for Education and the Gini Index, all factors were influential on the rate of COVID-19 spread before and after stay-home orders. However, after lifting the orders, six of the factors were not influential on the rate of spread; these included: African-Americans, Population Density, Single Parent Households, Average Daily PM2.5, HIV Prevalence Rate, and Home Ownership. It was concluded that different factors from the ones controlling the initial spread of COVID-19 are at play after stay-home orders are lifted.

**KEY MESSAGES**
- Observed rates of COVID-19 infection at the County level in the U.S. are not directly associated with adoption and lifting of stay-home orders.
- Disadvantages in sociodemographic determinants negatively influence the rate of COVID-19 spread.
- Counties with more young individuals, racial and ethnic minorities, and higher population densities have greater difficulty suppressing transmission.

## 1. Introduction

Accounting for ∼17% of all global COVID-19 cases, as of March 2022, the U.S. has an estimated 79.6 million infections, of which almost 969,000 have died [1]. At the beginning of the pandemic, before current advances in developing vaccines and in the absence of proven treatments, federal and state governments instituted stay-home orders to slow the rate of infection. However, population lockdowns (lockdowns, stay-home, stay-home order and order were used as interchangeable terminologies throughout the manuscript) and social distancing measures, while critically important, are not the norm in American society. In disadvantaged communities, it is more likely to have: more people that may be unable to physically distance or use personal protection equipment [2], higher rates of chronic comorbidities, poorer access to care and increased exposure at work; all of which has led to disproportionate rates of infection and greater mortality within communities of colour. Minorities and youth, for example, are at higher risk of being exposed to COVID-19 because their occupations require them to work outside of their homes [3]. Jobs in transportation, manufacturing, sales and agriculture often cannot be performed remotely and require close contact with customers or team members [4]. Furthermore, prior studies have demonstrated the prevalence of COVID-19 infections and adverse outcomes associated with underlying medical conditions [5], exposure to environmental pollution [6], and social determinants [7]. Thus, in a pandemic such as COVID-19, understanding who is at risk of infection, morbidity and mortality throughout the various stages of a pandemic is a critical consideration.

This paper examines the national daily reported case data to understand their longitudinal variability at the county level and to associate the observed rates of infection with the adoption of mitigation policies such as lockdowns and social distancing. Importantly, the study controls for social and economic risk factors, physical environment and health behaviours to elucidate the social determinants contributing to the observed rates of infection. The study objectives include: 1) categorizing counties based on the severity of the pandemic; 2) identifying social determinants that are significantly correlated to increasing cases; 3) undertaking a longitudinal analysis of weekly slopes and their progression before and during the issuance of stay-home orders, and after their lifting; 4) elucidating underlying relationships between weekly disease spread slopes and social determinants; and 5) understanding the disparate effectiveness of the stay-home orders in the various counties.

## 2. Methods

### 2.1. Data acquisition and processing

COVID-19 case number data at the county level from 22 January 2020 till 1 June 2020 [1] were used and were normalized to the 2018 county population data [8] (Figure S1 in the Supplementary Information (SI)). All of the analyses in this study are based on the Ecosocial Theory and to satisfy its critical constructs, including 1) embodiment, 2) pathways to embodiment, 3) the cumulative interplay of exposure, susceptibility, and resistance, and 4) agency and accountability. The choice of *Population Determinants* or PDs was based on embodiment and pathways to embodiment to consider historical, political, economic, temporal and spatial effects and assuming that the selected variables might have an effect on the spread behaviour of COVID-19. The selected variables to satisfy embodiment include underlying health conditions (e.g. HIV prevalence rate, diabetes, obesity, average number of physically and mentally unhealthy days, fair or poor health and disability), while the variables for pathways to embodiment include public health variables (e.g. life expectancy, age-adjusted death rate, health insurance, food insecurity, and primary care physicians ratio), sociodemographic variables (e.g. race, ethnicity and age), economic (income, Gini Index, public assistance, and homeowners) and behavioural determinants (e.g. drinking, smoking and physical activity). In addition, other variables such as employment rate (represented as “Unemployed” in Table 1), population and education could be a barrier to social distancing. Thus, with a lower percentage of unemployment and education rates and higher population density in a county, one can expect a high rate of contagion. Health outcomes and social determinants data, consisting of health behaviours, clinical care, social, economical and physical environment measures, were obtained from the Robert Wood Johnson Foundation [11]. Agency and accountability were considered via the local and federal governments' intervention. Such intervention was mainly through stay-home orders. The dates for the issuance and lifting of stay-home orders were compiled from Cable News Network (CNN) and National Broadcasting Company (NBC) [12,13]. For most states, uniform dates were assigned to the entire state. However, for some counties, such as Davis County and Salt Lake County in Utah, the dates were different from the rest of the state. Table 1 lists all variables (sociodemographic-economic-health-physical-ambient parameters referred to as PDs).

**Table 1.** Name and a brief description of all variables (Population Determinants or PDs) used in the study.

Variable	Description
Adults with Diabetes [13]^a^	Percentage of adults aged 20 and above with diagnosed diabetes
Adults with Obesity [9]	Percentage of the adult population (age 20 and older) that reports a body mass index (BMI) greater than or equal to 30 kg/m2
African-American [10]	Portion of population who are African-Ameican (%)
Age-Adjusted Death Rate [9]	Number of deaths among residents under age 75 per 100,000 population (age-adjusted)
Average Daily PM_2.5_ [9]	Average daily density of fine particulate matter in micrograms per cubic metre (PM_2.5_)
Average Number of Mentally Unhealthy Days [9]	Average number of mentally unhealthy days reported in past 30 days (age-adjusted)
Average Number of Physically Unhealthy Days [9]	Average number of physically unhealthy days reported in past 30 days (age-adjusted)
Disability [10]	Portion of population with at least one disability (%)
Education [10]	Portion of the population older than 25 years old with high school diplomas or higher degrees. (%)
Excessive Drinking [9]	Percentage of adults reporting binge or heavy drinking (%)
Fair or Poor Health [9]	Percentage of adults reporting fair or poor health (age-adjusted)
Food Insecure [9]	Percentage of population who lack adequate access to food (%)
Gini Index [10]	A measure of statistical dispersion in income or wealth distribution
Health Insurance [10]	Portion of population with at least one health insurance (%)
Hispanic [10]	Portion of population who are hispanic (%)
HIV Prevalence Rate [9]	Number of people aged 13 years and older living with a diagnosis of human immunodeficiency virus (HIV) infection per 100,000 population
Homeowners [9]	Percentage of occupied housing units that are owned
Life Expectancy [9]	Average number of years a person can expect to live
Median Age [10]	Median age of the population
Median Income [10]	Median income (adjusted to 2018 U.S. dollars)
Over45 [10]	Portion of population with a age ≥45 years old (%)
Over60 [10]	Portion of population with a age ≥60 years old (%)
Physically Inactive [9]	Percentage of adults age 20 and over reporting no leisure-time physical activity
Population Density [10]	Total population living within a square kilometre of a county
Primary Care Physicians Ratio [9]	Ratio of population to primary care physicians
Public Assistance [10]	Portion of the population who are part assistance programs that provide cash or in-kind benefits
Single Parent Households [9]	Percentage of children that live in a household headed by single parent (%)
Smokers [9]	Percentage of adults who are current smokers (%)
Social Association Rate [9]	Number of membership associations per 10,000 population
Unemployed [9]	Percentage of population ages 16 and older unemployed but seeking work
White [10]	Portion of population who are white (%)

^a^The data listed in this table was obtained from the source numbers shown here that could be found in the References section.

Non-parametric (NP) statistical analyses were conducted since none of the variables were normally distributed across counties based on a Kolmogorov–Smirnov normality test. Although the Spearman's correlation analysis shows some significant correlations among the selected variables, the correlation was not strong enough (ρ < 0.6) to eliminate the correlated variables, and all variables were used.

### 2.2. Statistical analyses

The cumulative interplay of exposure, susceptibility and resistance was examined by using two different statistical analyses. These analyses were conducted to examine relationships between the spread of COVID-19 and PDs. First, a longitudinal analysis of the weekly slope of spread (increase in the number of normalized cases in a week divided by seven days) was calculated for a 10-week period. Second, the effectiveness of stay-home orders was examined, and different COVID-19 spread behaviour within counties before, during and after the order was analysed. To be able to compare the magnitude of various correlation coefficients, the Fisher Z-transformation (shown as z′ in the results section**)** was used to transform the sampling distribution of correlation coefficients to z-scores.

#### 2.2.1. Slope of the COVID-19 spread over time

"DAY1" was defined as the first time a non-zero COVID-19 case was reported. The slope of the cumulative spread curve was calculated each week for 10 weeks after DAY1. The analysis was conducted for counties with non-zero normalized cases as of 1 June 2020, available data for at least eight weeks since DAY1, and that had non-zero slopes for at least three intervals out of 10, resulting in a total of 2292 counties. The nationwide median weekly slopes were calculated in SPSS.

The number of days from DAY1 till the issuance and lifting dates of the order was calculated for each county. Median slopes were estimated to provide a snapshot of the order effectiveness. Nationwide medians were also calculated as well as nationwide weekly slopes to obtain a nationwide longitudinal trend of weekly slopes. The aforementioned analyses were repeated using 14-day hindsight moving averaged normalized number of cases to obtain a smoother spread curve.

Independent samples Kruskal–Wallis Test was performed to determine whether the slopes of spread within different weeks are different from each other. Non-parametric correlation analyses among the weekly slopes and the PDs were conducted. The longitudinal change in the order of determinants with the strongest significant correlation with the weekly slopes was used to identify the most influential PDs of COVID-19 spread behaviour. Radar (spider) charts were developed to illustrate longitudinal changes in the PD order and the relative magnitude of their influence over time.

#### 2.2.2. Effect of stay-home orders on the spread of COVID-19

Two ratios were defined to capture the effectiveness of issuing the Orders and the potential changes in the slope of spread after lifting the Orders:

$$\mathrm{Ratio}\;1\;=\frac{\mathrm{Slope}\;of\;\mathrm{the}\;\mathrm{cumulative}\;\mathrm{curve}\;\mathrm{from}\;\mathrm{Stay}\;\mathrm{Home}\;\mathrm{order}\;\mathrm{till}\;it\;\mathrm{ends}}{\mathrm{Slope}\;of\;\mathrm{the}\;\mathrm{cumulative}\;\mathrm{curve}\;\mathrm{from}\;\mathrm{first}\;\mathrm{confirmed}\;\mathrm{case}\;to\;\mathrm{Stay}\;\mathrm{Home}\;\mathrm{order}\;}\mathrm{Ratio}\;2=\frac{\mathrm{Slope}\;of\;\mathrm{the}\;\mathrm{cumulative}\;\mathrm{curve}\;\mathrm{from}\;\mathrm{the}\;\mathrm{end}\;of\;\mathrm{Stay}\;\mathrm{Home}\;\mathrm{order}\;to\;\mathrm{June}\;1st}{\mathrm{Slope}\;of\;\mathrm{the}\;\mathrm{cumulative}\;\mathrm{curve}\;\mathrm{from}\;\mathrm{Stay}\;\mathrm{Home}\;\mathrm{order}\;\mathrm{till}\;it\;\mathrm{ends}\;}$$

A Ratio 1 greater than one indicates that the issuance of the stay-home order was probably late because COVID-19 was spreading faster after the issuance compared with the time before the order, and vice versa. Based on Ratio 1, counties were categorized using a 3-tiered classification as:

- "Lower slope after the issuance of the stay-home order",
- "Higher slope after the issuance of the stay-home order," and
- "First case happened during the stay-home order."

A value greater than one for Ratio 2 means lifting the stay-home order was associated with an increase in the number of cases. Counties were categorized into two categories using Ratio 2:

- "Lower slope after lifting the stay-home order", and
- "Higher slope after lifting the stay-home order"

Mann–Whitney U test was used to find significant differences in the values of PDs among the defined "Higher" and "Lower" groups in Ratio 1 and 2. The results were used to identify the PDs that were influential in terms of changing the slope of the spread curve during Stay-Home or lifting of the Order periods.

## 3. Results

### 3.1. Cross-sectional statistical findings

The normalized COVID-19 cases as of 1 June 2020 showed the highest correlation (Table S1 in the SI) with racial determinants African-American (z′= +0.54) and White (z′=-0.47) followed by population density (z′= +0.43), HIV prevalence rate (z′= +0.43) and age-related variables. Although race showed a very significant effect consistent with other studies [9,10,14], the percent Hispanic (z′= +0.17) showed a significant but less strong impact on normalized case numbers. Spearman's correlation coefficient for the percent older than 60 and 45 years old was −0.36 and −0.32, respectively and 0.31 for median age.

The HIV prevalence rate (z′= +0.43) showed the highest correlation, followed by disability (z′= −0.21) and adults with obesity (z′= +0.13). All other factors either showed a significant but weak correlation or no significant correlations. Average daily particle matters smaller than 2.5 µm (PM_2.5_ with a z′= +0.32) showed significant positive correlations verifying the effect of air quality [15]. Single-parent households (z′= +0.25) and education (z′= −0.15) showed a strong significant correlation with normalized cases, whereas social association rate showed a significant but weak correlation.

The median income showed a positive but weak correlation with the number of cases (z′= +0.10). However, The Gini Index showed a positive and stronger correlation with the total normalized cases (z′= +0.18), reflecting the difference in living cost standards among the studied counties. Homeownership (z′= −0.20) showed a significant correlation, whereas the portion of the population on public assistance and unemployed had a significant but weak correlation; children in poverty showed no significant correlation (*p*-value >.05).

### 3.2. Slope analysis

#### 3.2.1. Weekly spread slopes

**Figure 1.**
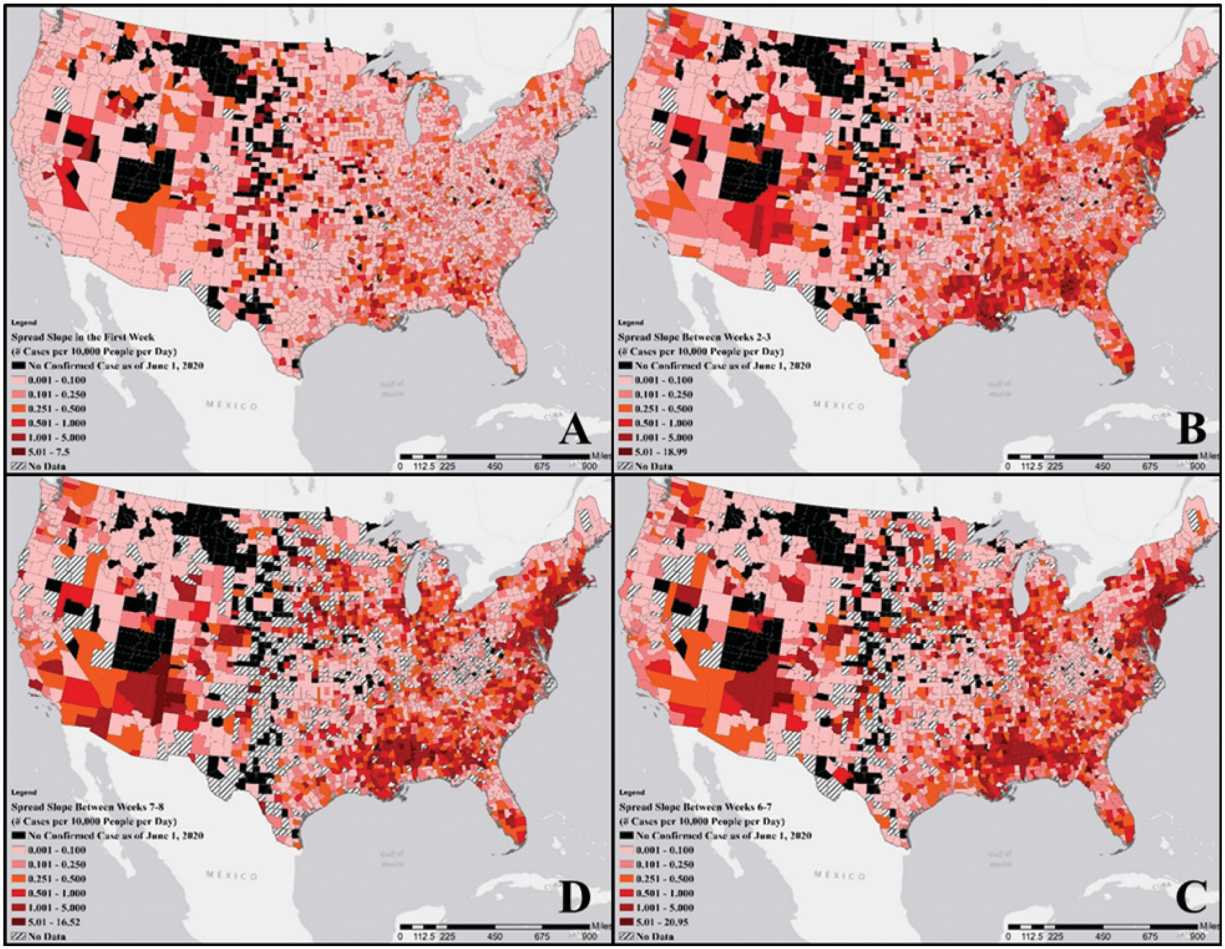
Geospatial distribution of weekly slopes (number of cases per 10,000 people per day) of the COVID-19 spread curve from DAY1 (first day with a reported non-zero confirmed case): (A) at the end of the first week, (B) from the end of week 2 to the end of week 3, (C) from the end of week 5 to the end of week 6, and (D) from the end of week 7 to the end of week 8.

Figure 1 shows the weekly slopes of the COVID-19 spread curve for the first week (W0-1), W2-3, W5-6 and W7-8 since the first observed case for each county (see Figure S2 through S11 in the SI). Although the states located on the West Coast and in the southwestern part of the U.S showed continuous high weekly slopes, those located on the East Coast and in the Midwest showed fluctuating weekly slopes. The results of the Independent Samples Kruskal–Wallis test rejected the null hypothesis indicating that the distribution of the slope was the same across the different weeks (*p*-value≪.05). The pairwise Mann–Whitney U test showed significant differences among the slopes in the first week (W1) and W2 with all other weeks, whereas the slope between weeks 2 and 3 (W3) was only significantly different from W8, W9 and W10. Finally, slopes in weeks W4, W5, W6, W7 and W8 were not significantly different from each other, whereas they were different from W9 and W10.

**Figure 2.**
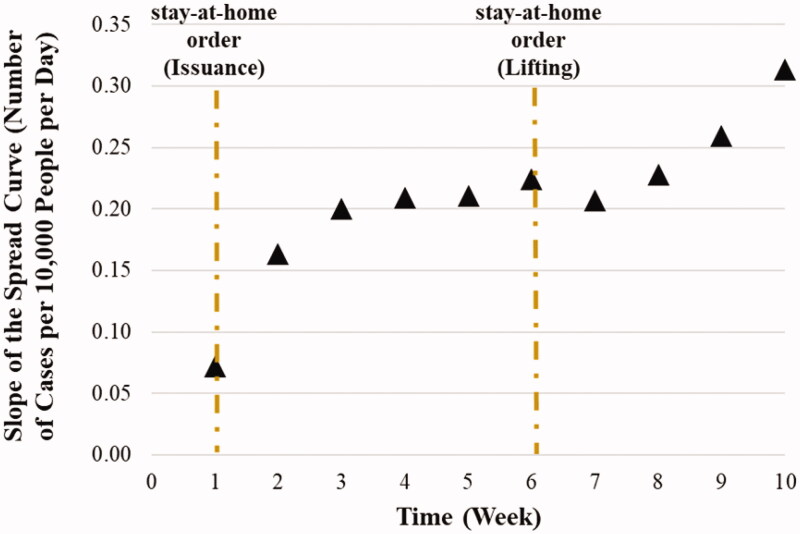
The longitudinal nationwide median of weekly slopes (number of cases per 10,000 people per day) using the normalized cases. The dashed lines represent the national median days since DAY1 for the issuance and lifting the stay-home order.

A similar pattern was observed in the median weekly slope values shown in Figure 2 and the box plot in Figure S12 in the SI: an initial rise in the weekly slope that starts to flatten between weeks 3 and 4, followed by another rise at the end of week 8. Using the 14-day hindsight moving average values (Figure S13 and 14 in the SI) led to similar results except that W2-3 was also significantly different from all other weeks and W4, W5, W6, W7, W8 and W9 showed a difference only when compared with W10. The nationwide medians for the number of days from DAY1 till the issuance and lifting dates of the Order were 7 and 43 days, respectively (the vertical dashed lines in Figure 2 and S14 in the SI). Considering the two-week incubation time for COVID-19, the issuance of the stay-home order at the end of week one resulted in flattening the slope rate at the end of week 3 and lifting it at the end of week 6 caused a rise in the weekly slope values starting at the end of week 8. However, it is noted that all 2292 counties studied in the slope analysis had continuous data for the first eight weeks since DAY1, but that number decreased to 1867, and 1099 counties for weeks 9 and 10, respectively.

**Figure 3.**
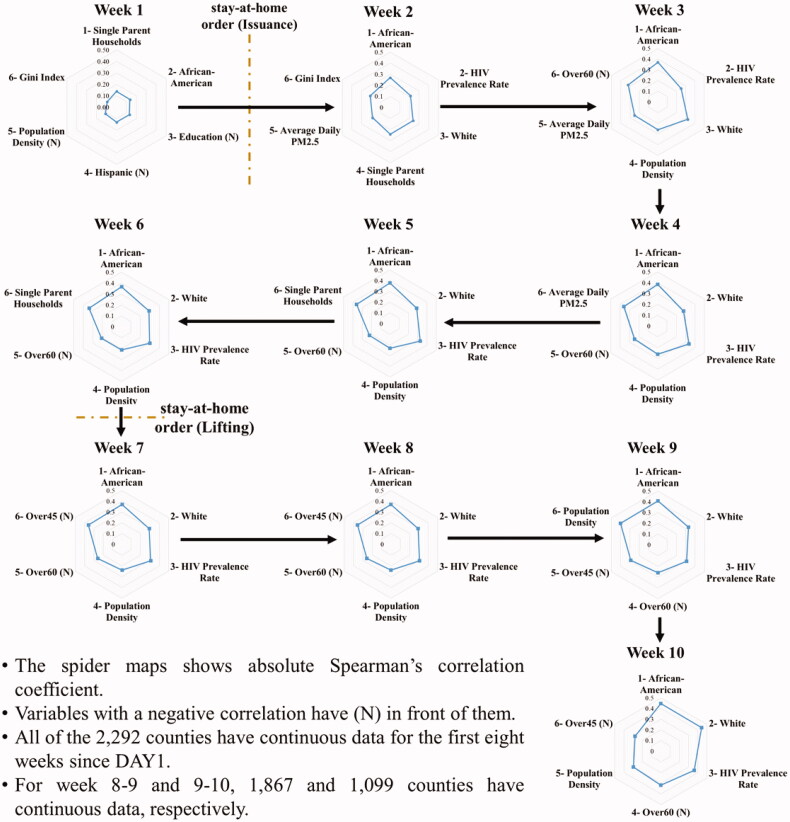
The top six most correlated PDs with the slope of the COVID-19 spread curve and corresponding Spearsman's correlation coefficients for different weeks.

The results of Spearman's correlation analysis within each time interval (weeks) revealed that different PDs had different levels of significance and effect on the weekly slopes. The first six determinants, with the most significant positive/negative correlation, as well as their correlation coefficient with the spread slopes, are shown in Figure 3 as are nationwide medians. All significant correlations were very weak in the first week, with single-parent households, African-American, education, Hispanic, Population density and Gini Index showing the highest correlations with the first week spread slope. Starting from the second week and beyond, placing in effect a stay-home order and allowing for the passage of two weeks, the effect of the Gini Index, education and Hispanic was replaced with white, HIV prevalence rate, Over60 and Average Daily PM_2.5_ rate till the end of week 4. At the beginning of week five, as shown in Figure 3, till the Order lifting time, single-parent households placed among the top six instead of Average Daily PM_2.5_. Right after the Order lifting (Beginning of Week 7), the Over45 overtook the single-parent households place till week 10. It is important to note that race, population density (except for the second week) and HIV prevalence rate (except for the first week) were always among the top six influential PDs, social and economical variables were more important at the very beginning of the pandemic, whereas age distribution became more important around the time when the order was lifted. Average daily PM_2.5_ showed importance only when the order was in effect (W2-W4).

#### 3.2.2. Stay-home order period

A Mann–Whitney U test showed counties located in states that did not issue an order ended up with a smaller number of normalized cases compared with the ones with the order. In addition, the slope of COVID-19 spread from day one to 1 June 2020 was not significantly different among counties with and without the order pointing to the importance of social determinants. Figures S15 through Figure S28 in the SI show the distribution of the 14 chosen influential determinants (with an absolute correlation coefficient greater than 0.15) using box plots. In counties located in states with no Order, similar to the counties with no confirmed COVID-19 cases, the population density (Figure S15) was significantly lower (*p*-value≪.05) than in other counties. Similarly, the rate of African-Americans (Figure S16) and Hispanics (Figure S18) were lower in these counties, whereas the White ratio (Figure S17) was higher. Figures S29 and S30 show the membership of different counties relative to Ratio 1 and Ratio 2, respectively (see Table S2 in the SI). Out of 2454 counties with data, only 247 showed a lower slope after the stay-home order.

For Ratio 2, 1289 counties showed lower slopes even though the order had been lifted (Table S2 in the SI). This was partially explained *via* the social determinants (Figures S15 through Figure S28 and Table S3). Twelve out of 14 determinants showed significant difference (*p*-value < .05) among counties with higher and lower slopes when the slopes were compared before the Order issuance with the after till lifting (Ratio 1); this number was only 7 for Ratio 2 (Order duration vs. after lifting). A total of seven determinants showed similar behaviour, whereas one of those was not significant among higher and lower slopes in both Ratio 1 and 2 classes (Gini Index). The six common determinants that showed different distributions were age-related (median age, Over 45 and Over 60), racial and ethnic (White and Hispanic), and disability. Education was the only variable that was influential for Ratio 2 but not Ratio 1. In contrast, African-Americans, Single Parent Households, Average Daily PM_2.5_, HIV Prevalence Rate, and Home Ownership were influential on Ratio 1 but not on Ratio 2.

## 4. Discussion

The findings in the cross-sectional statistical analyses confirmed that COVID-19 transmission (as of 1 June 2020) is catalysed by a higher population density [16] and greater person-to-person contact [17]. Furthermore, the correlation analyses among the normalized cases and the age variables confirmed that individuals younger than 20 years old exhibit lower rates of COVID-19 [18] and that the population between 18 and 45 years of age holds a higher proportion of mobility-based occupations [19]. Although older populations are more vulnerable to COVID-19 hospitalization and mortality [5], our findings indicate that counties with younger populations are more likely to exhibit rising epidemic curves.

As is shown in the results section, communities with higher proportions of African Americans and Hispanic Americans are more likely to experience rising epidemic curves as noted in other studies [20–24]. This double jeopardy—racial or ethnic minorities experiencing a higher burden of disease and continuing to experience a higher burden with rising curves—exacerbates long-standing health inequities. However, a more thorough investigation (with more detailed data) is required to understand whether the finding is explained by the over-representation of minorities in jobs that require concentration of individuals or because of higher rates of underlying health conditions among racial and ethnic minorities. These disparities permeate other health conditions, particularly chronic diseases, which may be contributing to a new wave of infection in minority populations [25] especially given the considerable 41% of frontline workers who are people of colour [26]. With a decreased risk of severe illness and increased likelihood of being asymptomatic [27], younger individuals may feel more comfortable leaving their homes and neglecting social distancing measures. In addition, young individuals more often work in industries and positions that require them to leave their homes thus, contributing to greater mobility [28] relative to older individuals who may be retired or able to work from home. These factors taken together point to the implications for a new wave that could overwhelm colleges and other institutions where younger persons are over-represented.

The fact that counties located in states without an Order showed a smaller number of normalized cases compared with the ones with the order could be explained through some of the Population Determinants. These counties have better conditions in air quality indicators, health, social, economic parameters and education rate. Thus, one could conclude that the rate of spread was low enough in these counties that there was no need to issue an Order. The low number of counties with a lower spread slope after the stay-home order could be interpreted as the issuance of the order occurred, whereas the COVID-19 growth was in the exponential phase [29], and such issuance might only slow the increase in the slope rate (second derivative). Furthermore, for 954 counties, the first confirmed case (DAY1) occurred after the issuance of the order. The fact that African-Americans, Single Parent Households, Average Daily PM_2.5_, HIV Prevalence Rate and Home Ownership were influential on Ratio 1 (Order duration) but not on Ratio 2 (after lifting the order), can be interpreted to mean that these variables are influential in the spread of COVID-19 irrespective of lifting stay-home orders but are not directly associated with the rate at which the spread occurs once the order is lifted, other factors are at play.

As anticipated by the ecosocial theory, the findings from the study indicate that demographics play a an influential role in affecting the number of normalized COVID-19 cases, the rate of rise in cases and the effectiveness of stay-home orders in slowing the spread and flattening the spread curve. These results inform policies to address the continuing surge of COVID-19 cases and to develop long-term interventions to reduce spread, particularly among those at greatest risk. Such interventions could happen at multiple different social systems levels. Future works based on the Ecosocial Theory could help to identify areas for potential intervention to further limit COVID-19 spread within populations. For instance, addressing the observed differences in the Gini coefficient would require structural level intervention, whereas some of the other variables could be more amenable to more proximal interventions that can occur at the individual, household or employer levels. These results also suggest the need to evaluate the consequences of varying levels of higher person to person interactions; a second or multiple waves will continue to occur if current mandates of social distancing and stay-home orders are not rigorously implemented [30,31].

A number of limitations should be considered when interpreting these findings. First, data were used from the Johns Hopkins Coronavirus Resource Centre, which aggregates publicly available sources. Given inconsistencies in testing across communities, these data may not accurately capture the true number of cases in a county on a given day or during a given week. Second, although these patterns are observed at the county level, it is important to understand their variation at the census tract level [32]. Third, additional predictors could be included such as, mobility, mode of transportation (public or private car), the percentage working in various sections (i.e. in transportation, manufacturing, sales and agriculture at the county level) and political party affiliation.

## 5. Conclusions

The findings from the study indicate that demographics play a powerful role in affecting the number of normalized COVID-19 cases, the rate of rise in cases and the effectiveness of stay-home orders in slowing the spread and flattening the spread curve. Counties with higher percentages of young individuals, racial and ethnic minorities and higher population densities have experienced greater difficulty suppressing transmission. With the exception of Education and the Gini Index, all factors were influential on the rate of COVID-19 spread before and after stay-home orders. However, after lifting the orders, six of the factors were not influential on the rate of spread; these included: African-Americans, Population Density, Single Parent Households, Average Daily PM_2.5_, HIV Prevalence Rate and Home Ownership. It was concluded that different factors from the ones controlling the initial spread of COVID-19 are at play after stay-home orders are lifted.

## Supplementary Material

Supplemental Material

## Data Availability

The authors confirm that the data supporting the findings of this study are available within the article and its supplementary materials.
