# Supplementary material for: Examining longitudinal disparities in COVID-19 prevalence in the U.S.: a county level growth rate perspective
Source: Ann Med. 2022 May 6;54(1):1277–86. doi: 10.1080/07853890.2022.2069852 (PMC9090380; doi:10.1080/07853890.2022.2069852)
Supplement: Supplemental Material [file IANN_A_2069852_SM0231.docx]

**Examining Longitudinal Disparities in COVID-19 Prevalence in the U.S.: A County Level Growth Rate Perspective**


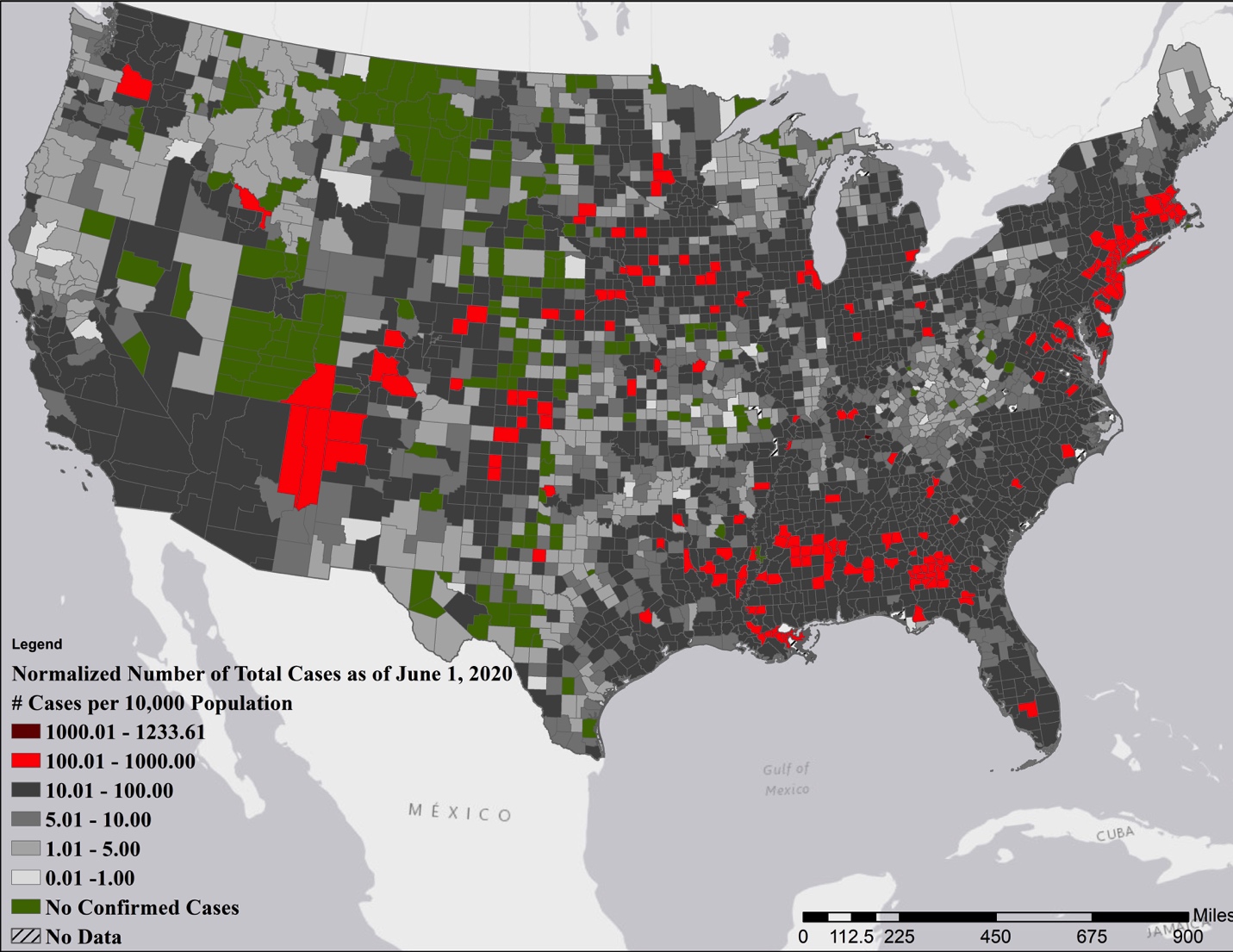


**Figure S1.** Distribution of the normalized total number of confirmed COVID-19 cases (defined as number of reported cases divided by the county population) at the county level within the contiguous U.S. as of June 1, 2020


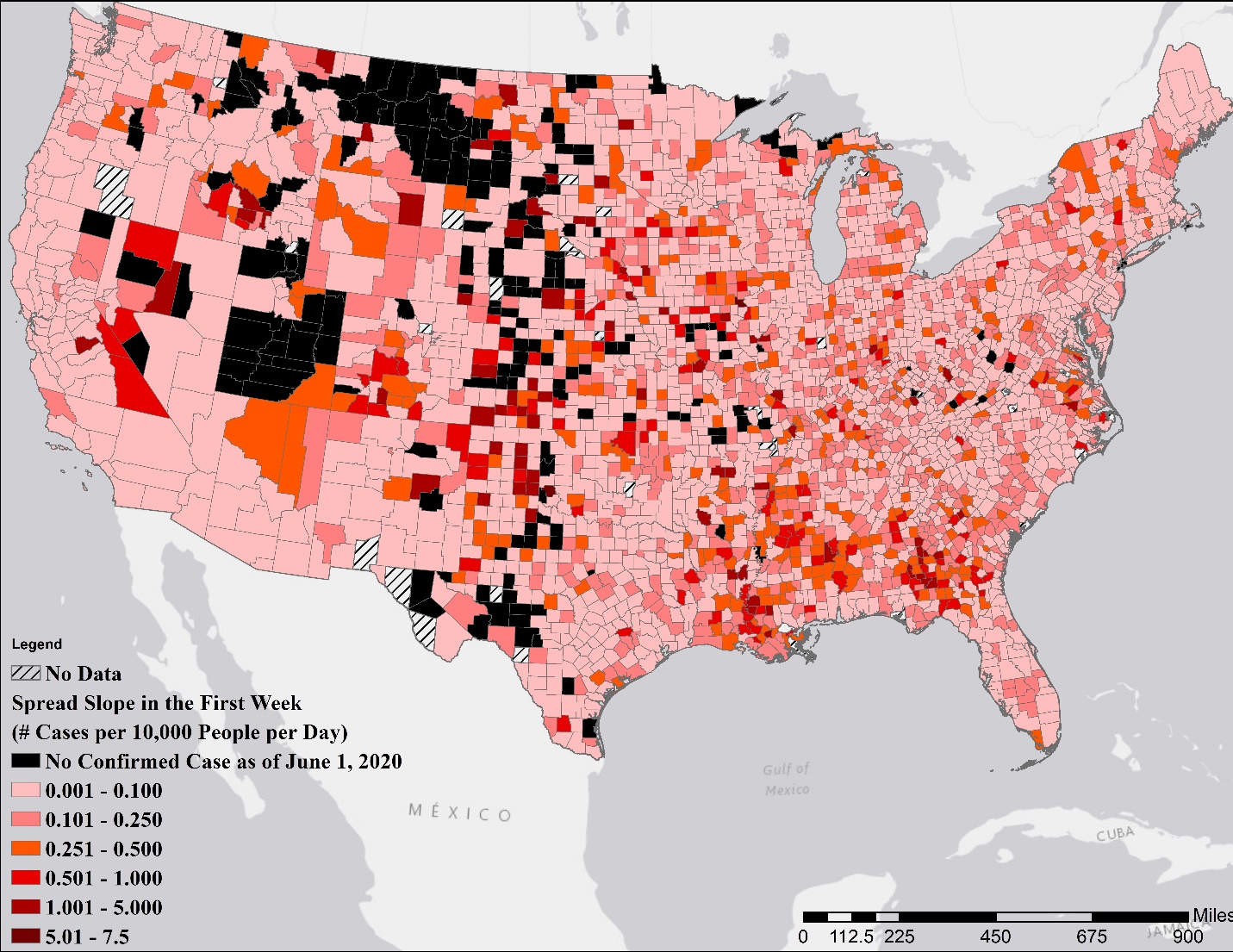


**Figure S2.** Geospatial distribution of weekly slopes (number of cases per 10,000 people per day) of the COVID-19 spread curve from DAY1 (first day with a reported non-zero confirmed case) to the end of the **first week** (DAY1 is not the same date for the counties)


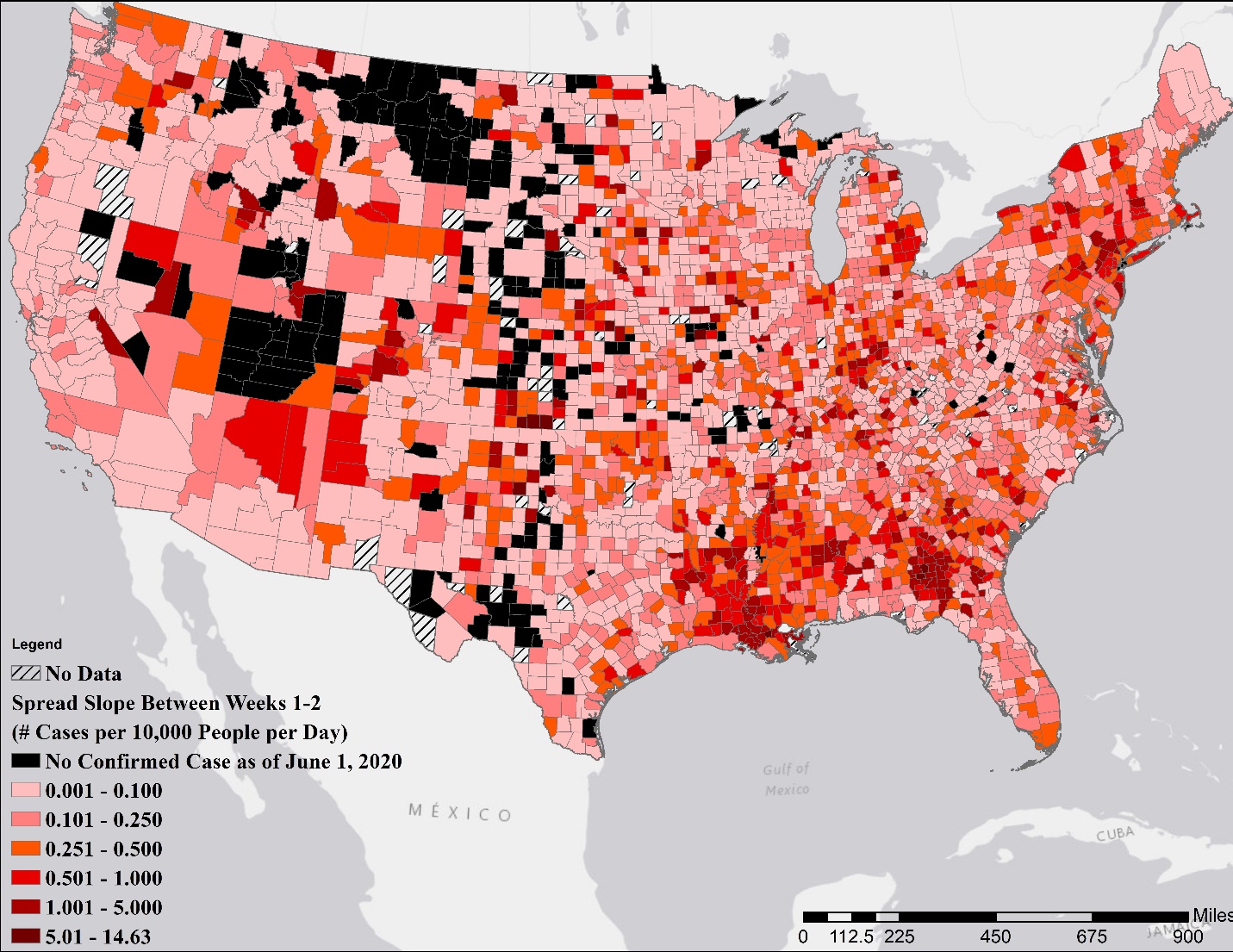


**Figure S3.** Geospatial distribution of weekly slopes (number of cases per 10,000 people per day) of the COVID-19 spread curve from the **end of week one to the end of week 2**


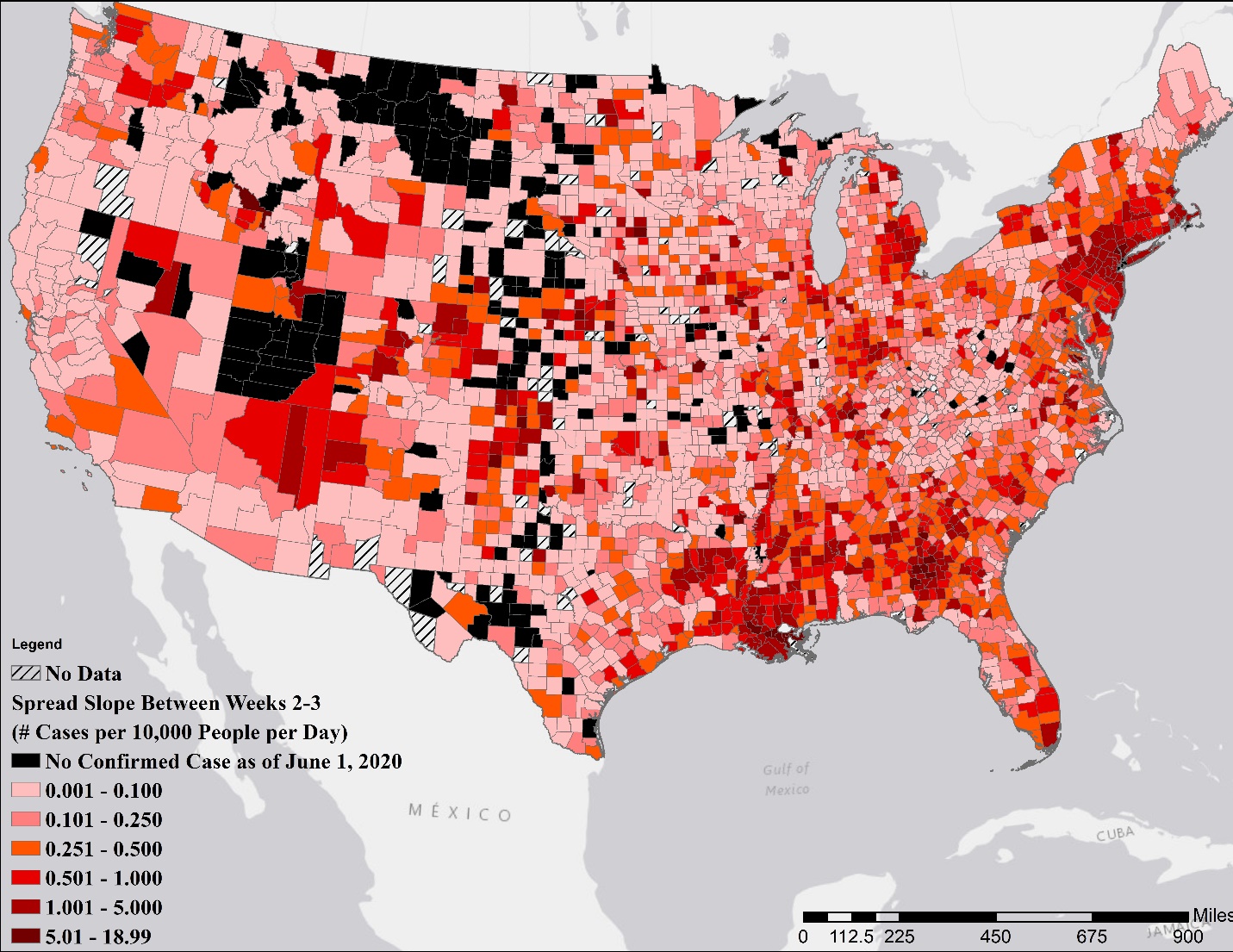


**Figure S4.** Geospatial distribution of weekly slopes (number of cases per 10,000 people per day) of the COVID-19 spread curve from the **end of week 2 to the end of week 3**


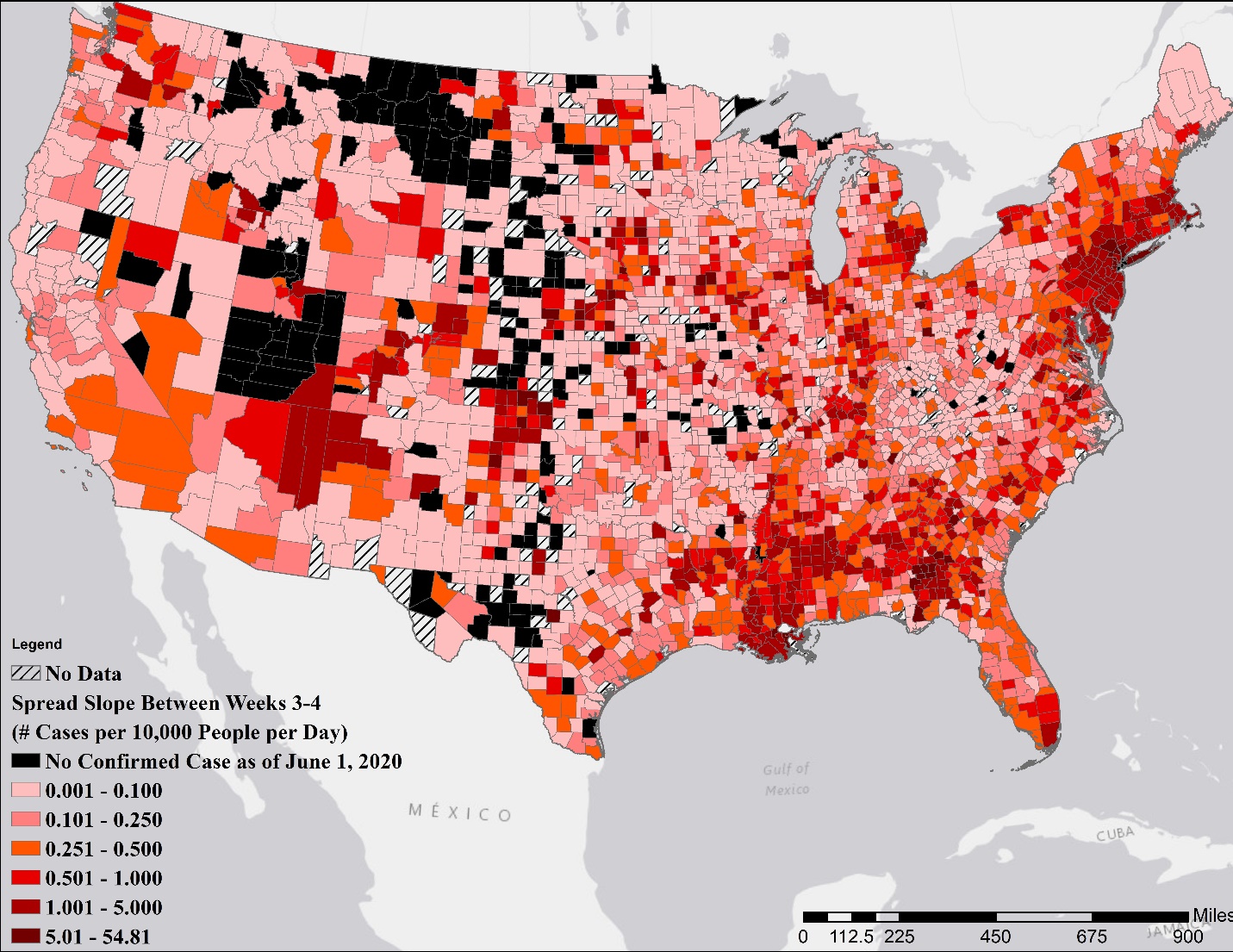


**Figure S5.** Geospatial distribution of weekly slopes (number of cases per 10,000 people per day) of the COVID-19 spread curve from the **end of week 3 to the end of week 4**


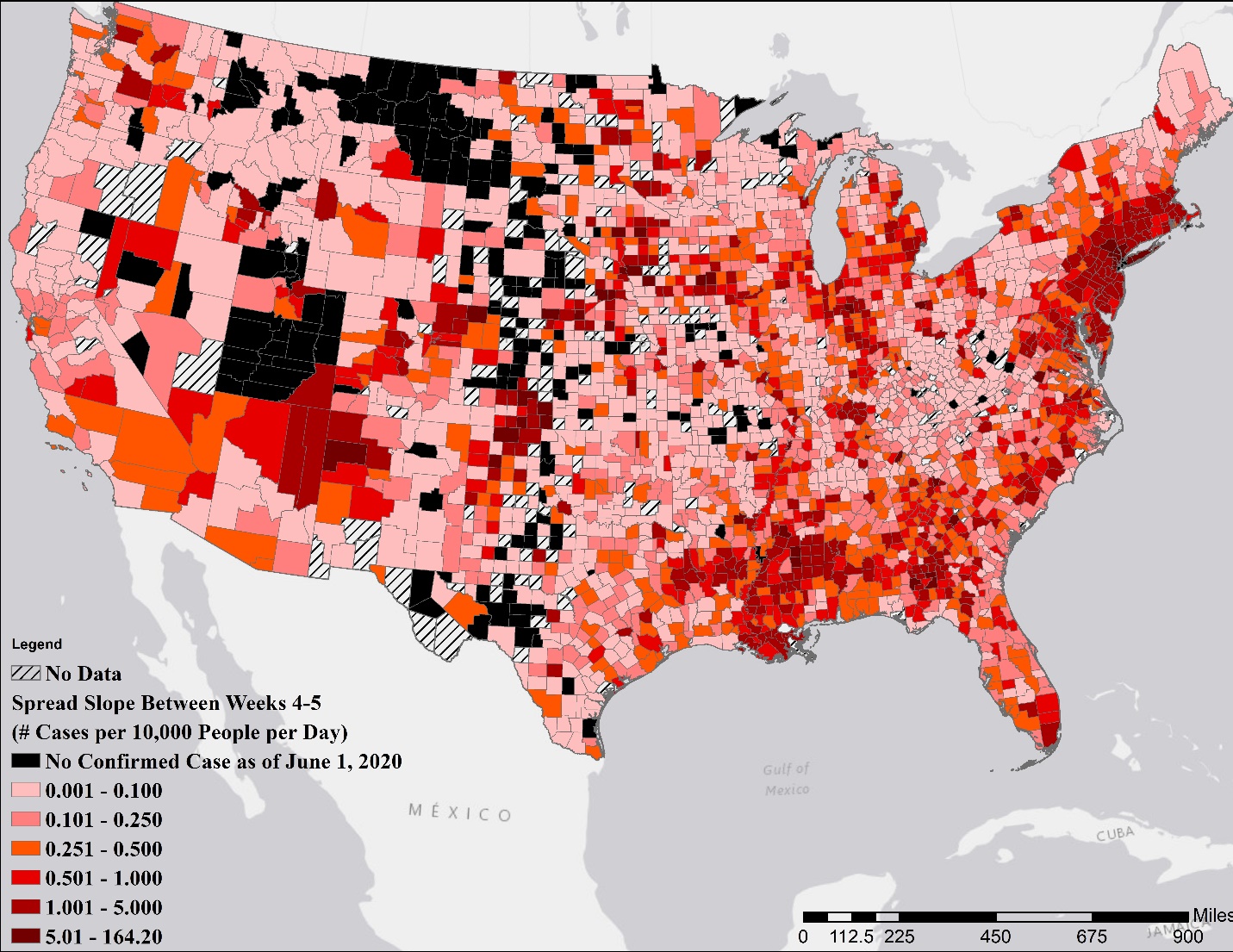


**Figure S6.** Geospatial distribution of weekly slopes (number of cases per 10,000 people per day) of the COVID-19 spread curve from the **end of week 4 to the end of week 5**


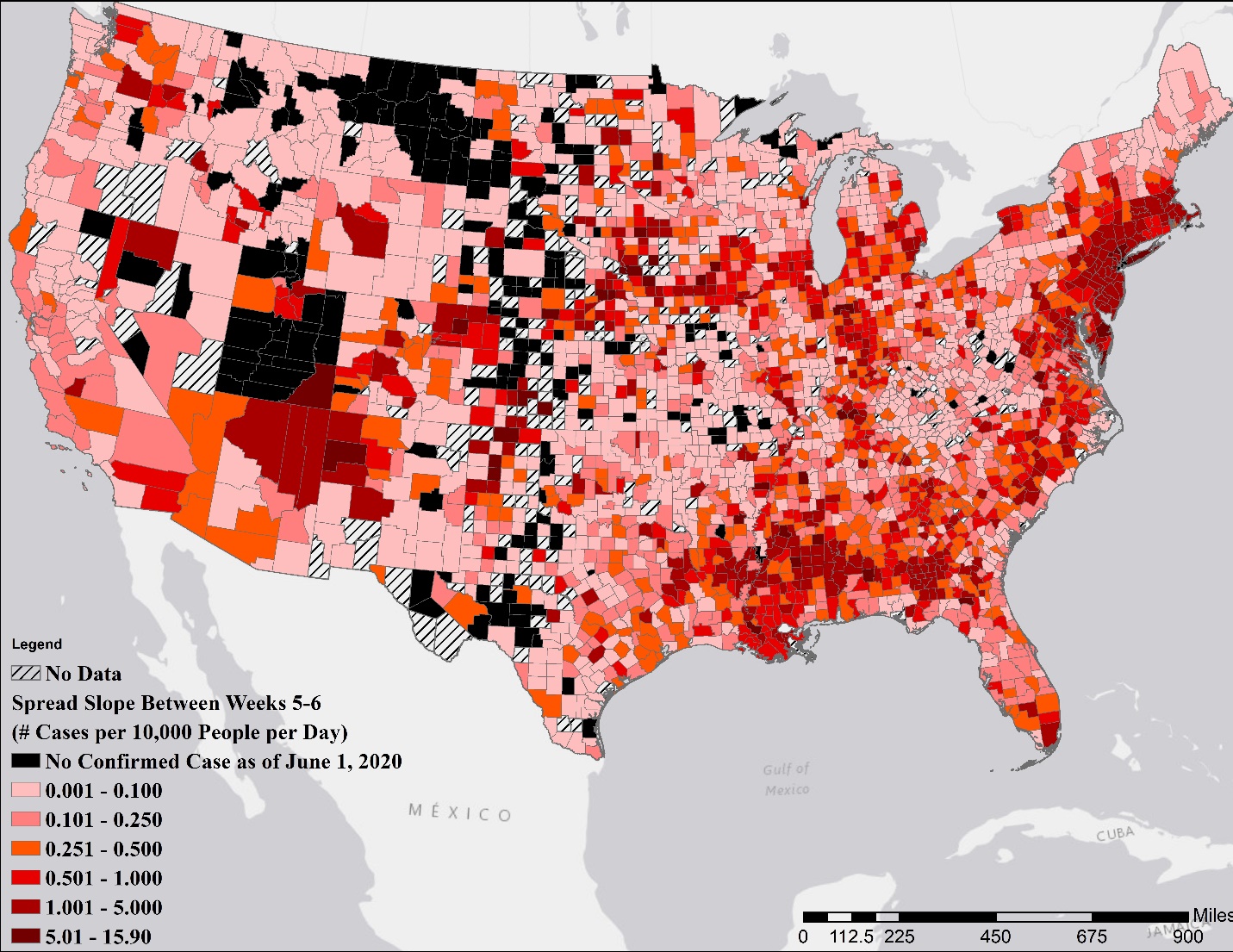


**Figure S7.** Geospatial distribution of weekly slopes (number of cases per 10,000 people per day) of the COVID-19 spread curve from the **end of week 5 to the end of week 6**


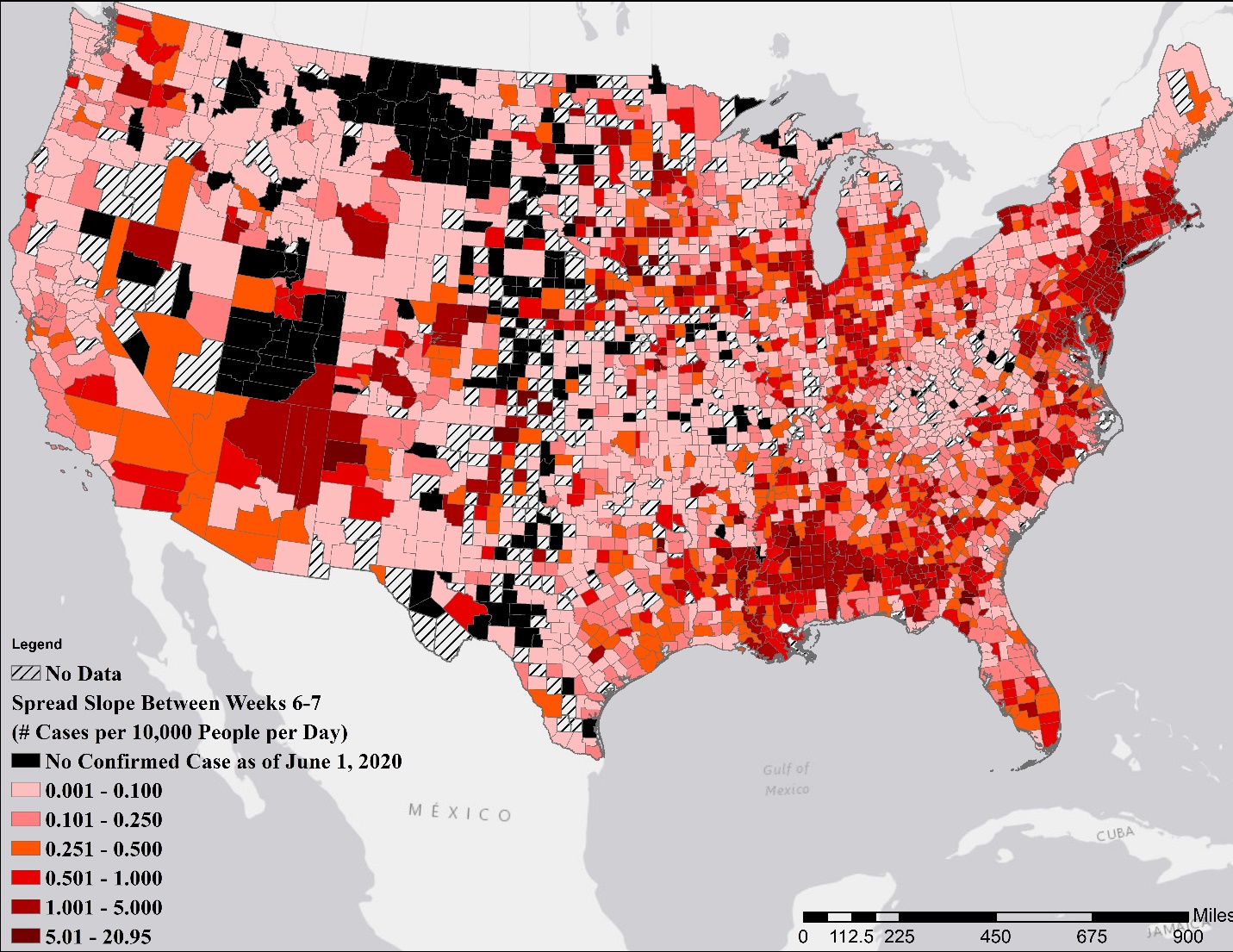


**Figure S8.** Geospatial distribution of weekly slopes (number of cases per 10,000 people per day) of the COVID-19 spread curve from the end of **week 6 to the end of week 7**


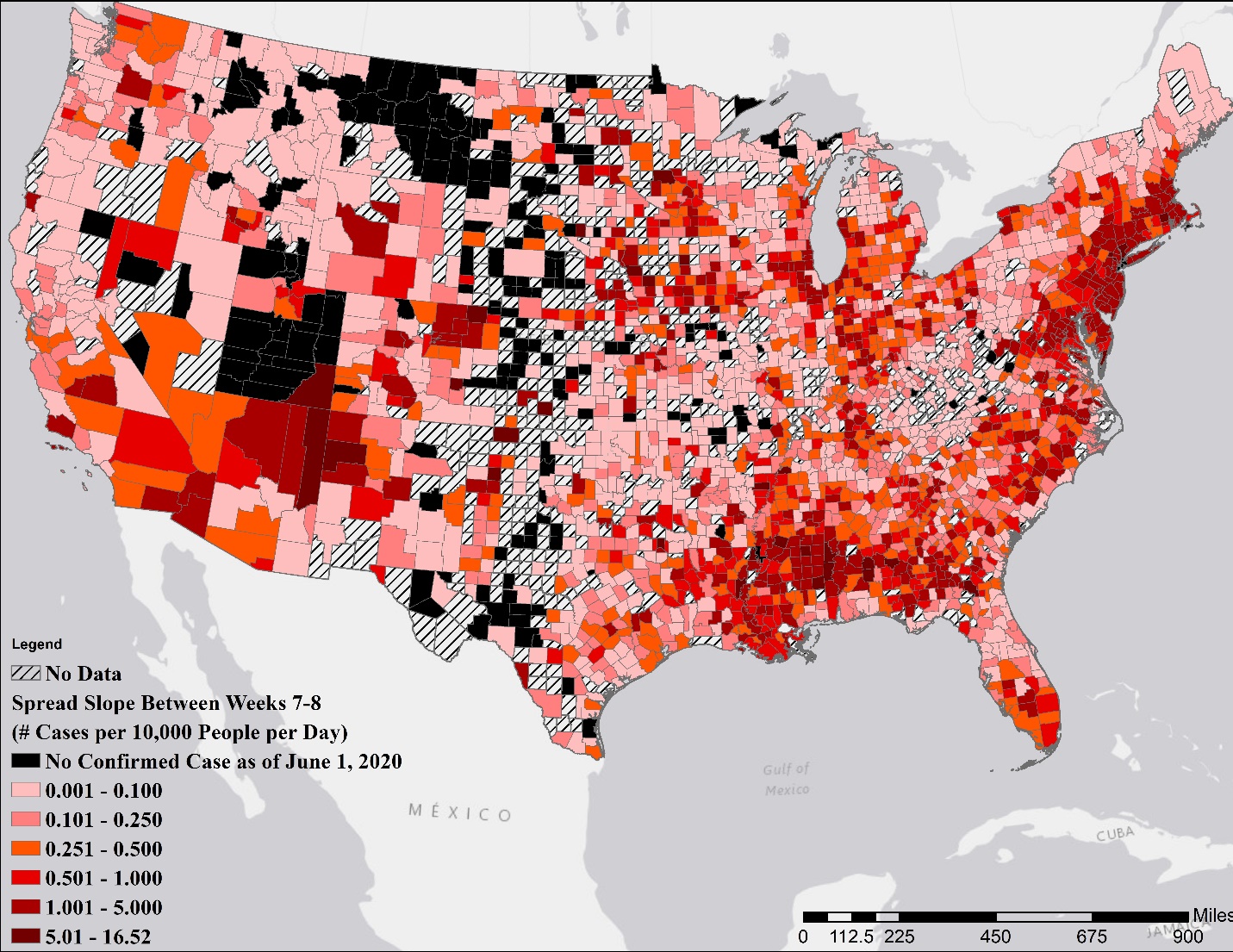


**Figure S9.** Geospatial distribution of weekly slopes (number of cases per 10,000 people per day) of the COVID-19 spread curve from the **end of week 7 to the end of week 8**


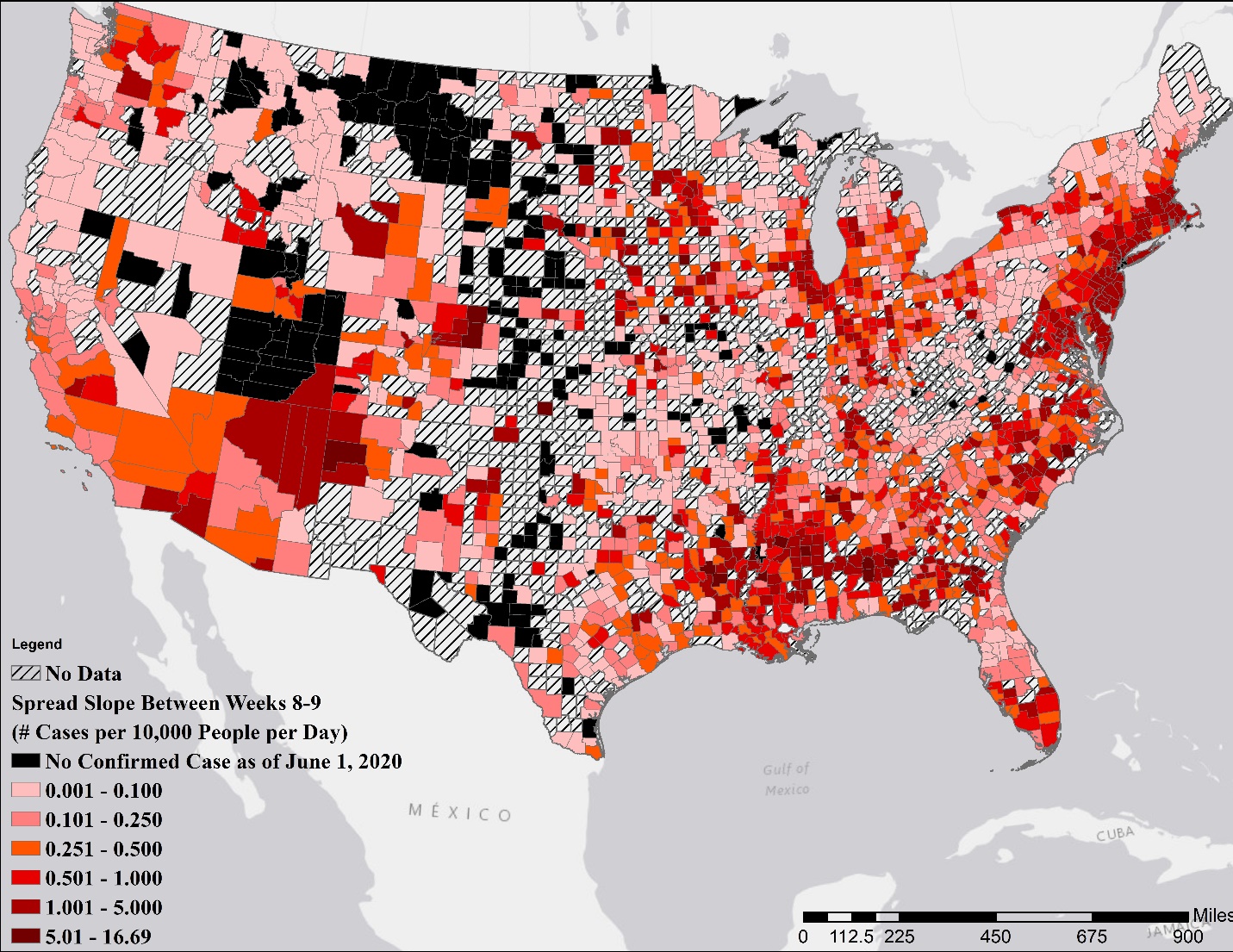


**Figure S10.** Geospatial distribution of weekly slopes (number of cases per 10,000 people per day) of the COVID-19 spread curve from the **end of week 8 to the end of week 9**


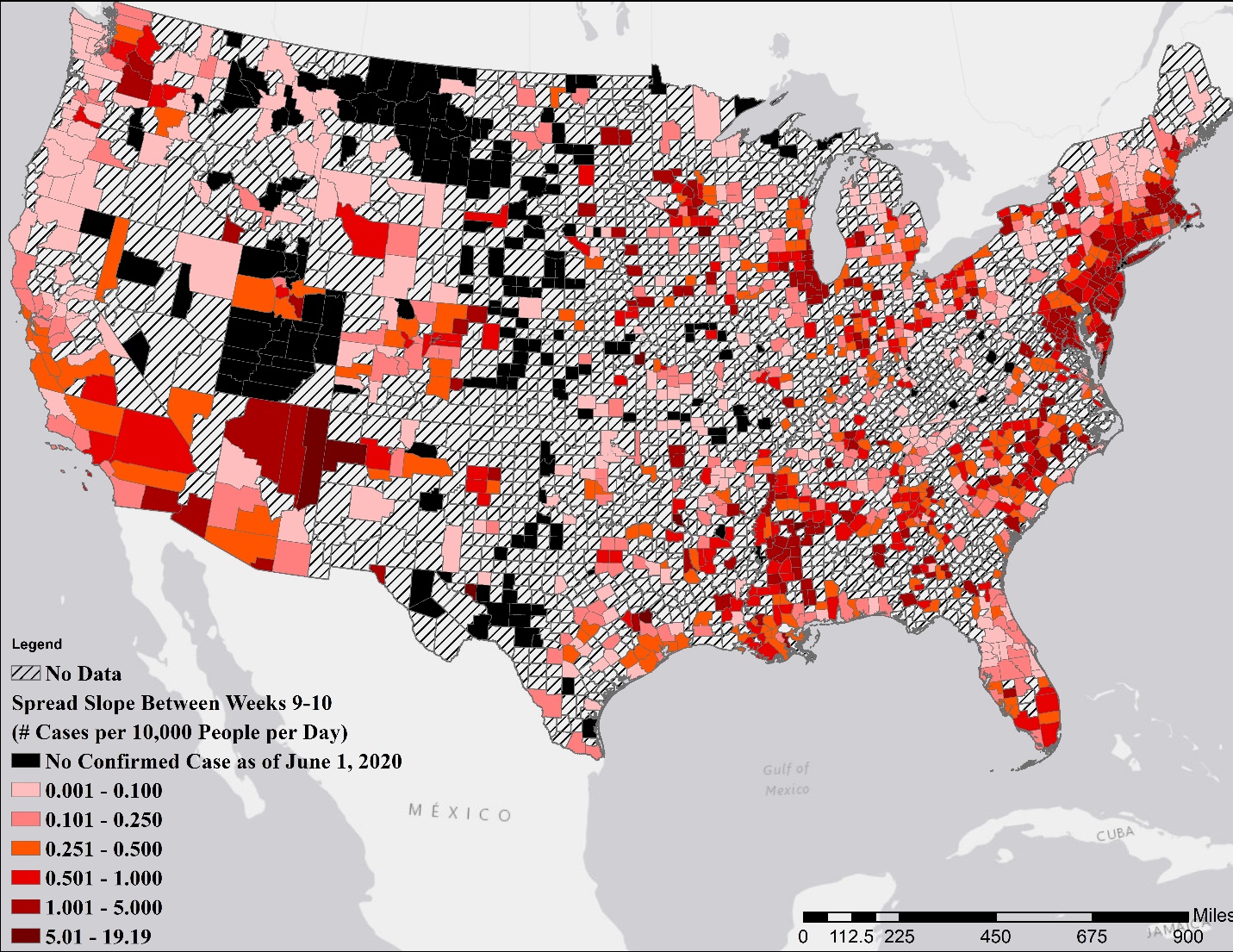


**Figure S11.** Geospatial distribution of weekly slopes (number of cases per 10,000 population per day) of the COVID-19 spread curve from the end of week 9 to the end of week 10.


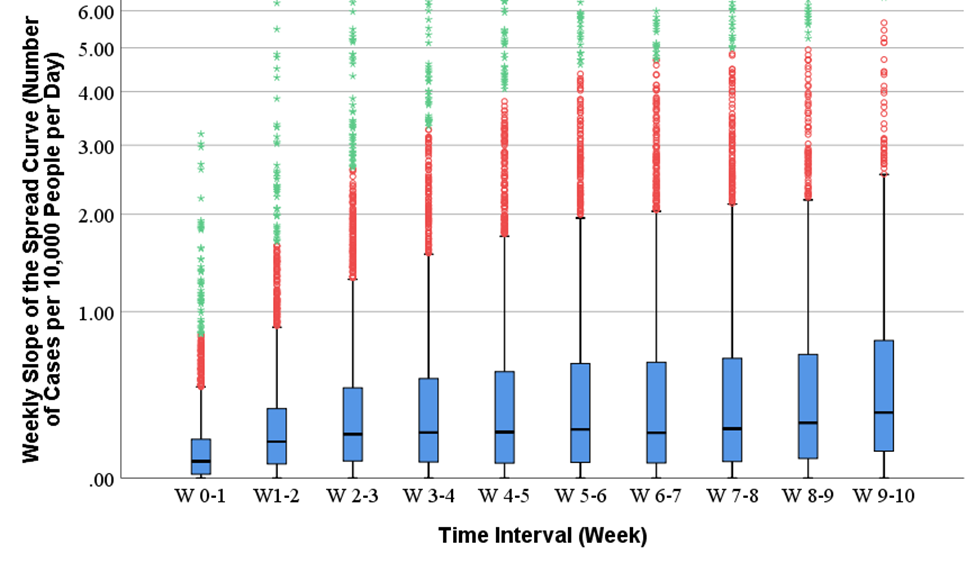


**Figure S12.** The longitundal nationwide box plot of weekly slopes (number of cases per 10,000 people per day) using the normalized cases


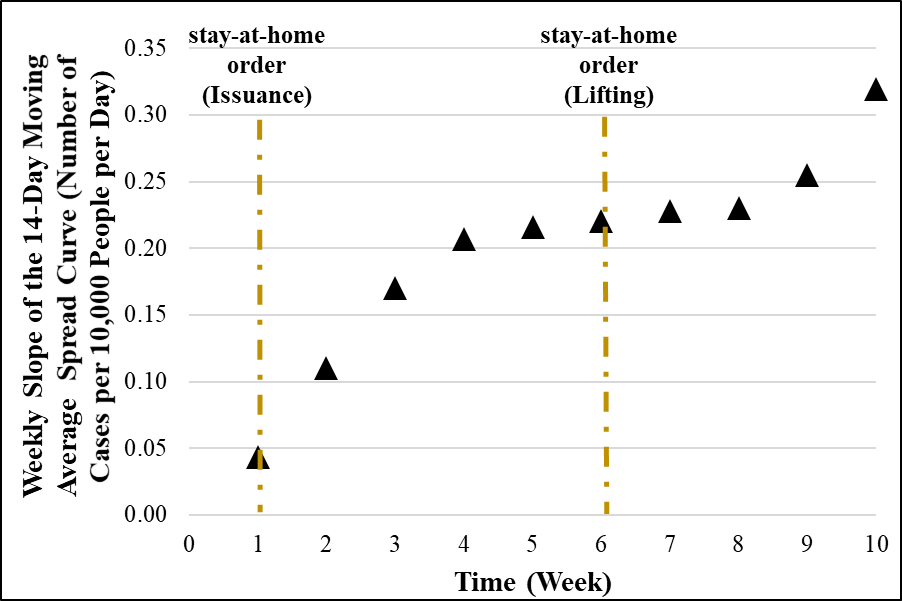


**Figure S13.** The longitudinal nationwide median of weekly slopes (number of cases per 10,000 people per day) using the 14-day backward moving average normalized cases. The dashed lines represent the national median days since DAY1 for the issuance and lifting the stay-at-home order .


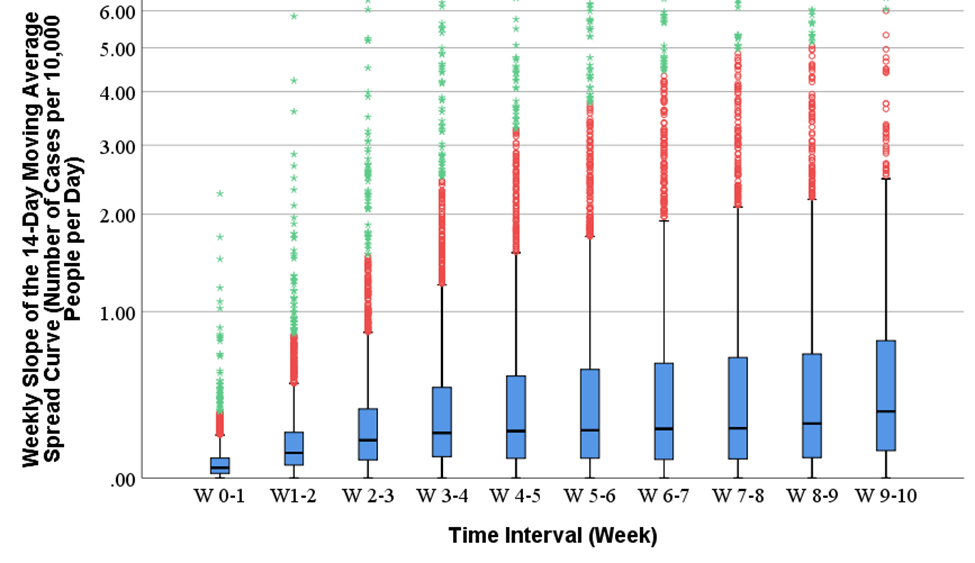


**Figure S14.** The longitudinal nationwide box weekly slopes (number of cases per 10,000 people per day) using the 14-day backward moving average normalized cases


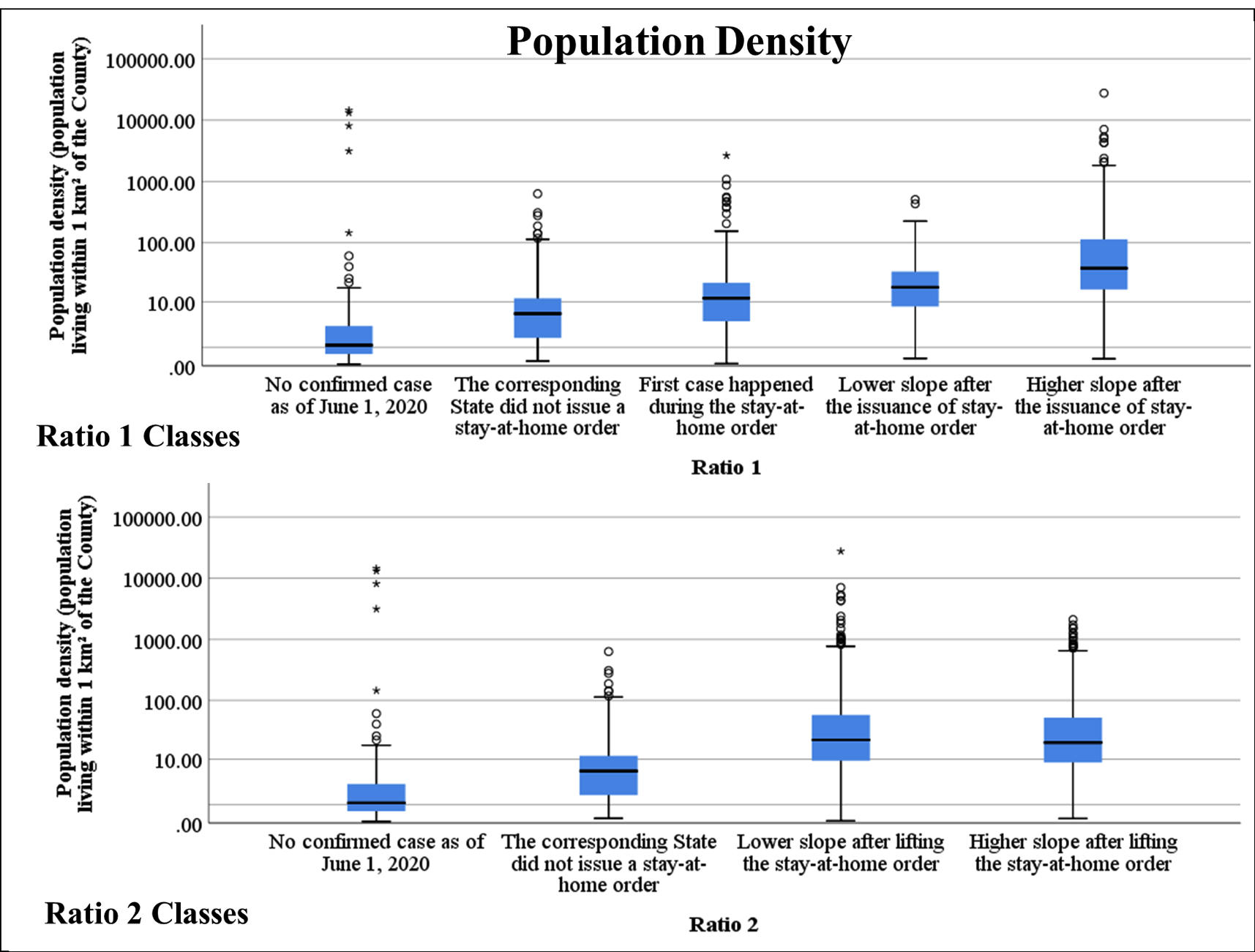


**Figure S15.** Distribution of population density determinant among different groups defined based on Ratio 1 and Ratio 2. Ratio 1 compares the slope of the spread curve before and after the issuance of the Stay-at-home order while Ratio 2 compares the slopes before and after lifting the Order.


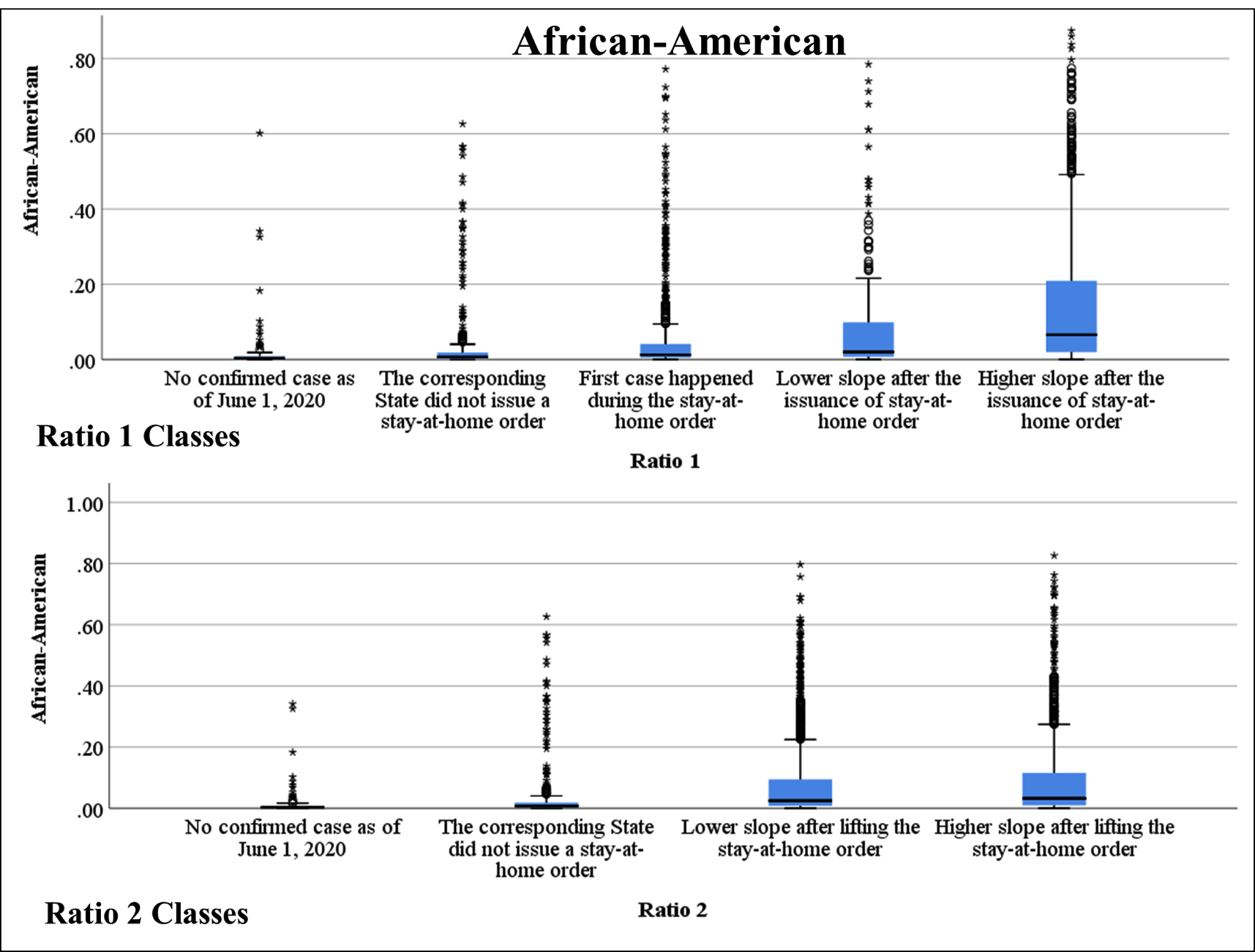


**Figure S16.** Distribution of African-American determinant among different groups defined based on Ratio 1 and Ratio 2. Ratio 1 compares the slope of the spread curve before and after the issuance of the Stay at Home Order while Ratio 2 compares the slopes before and after lifting the Order


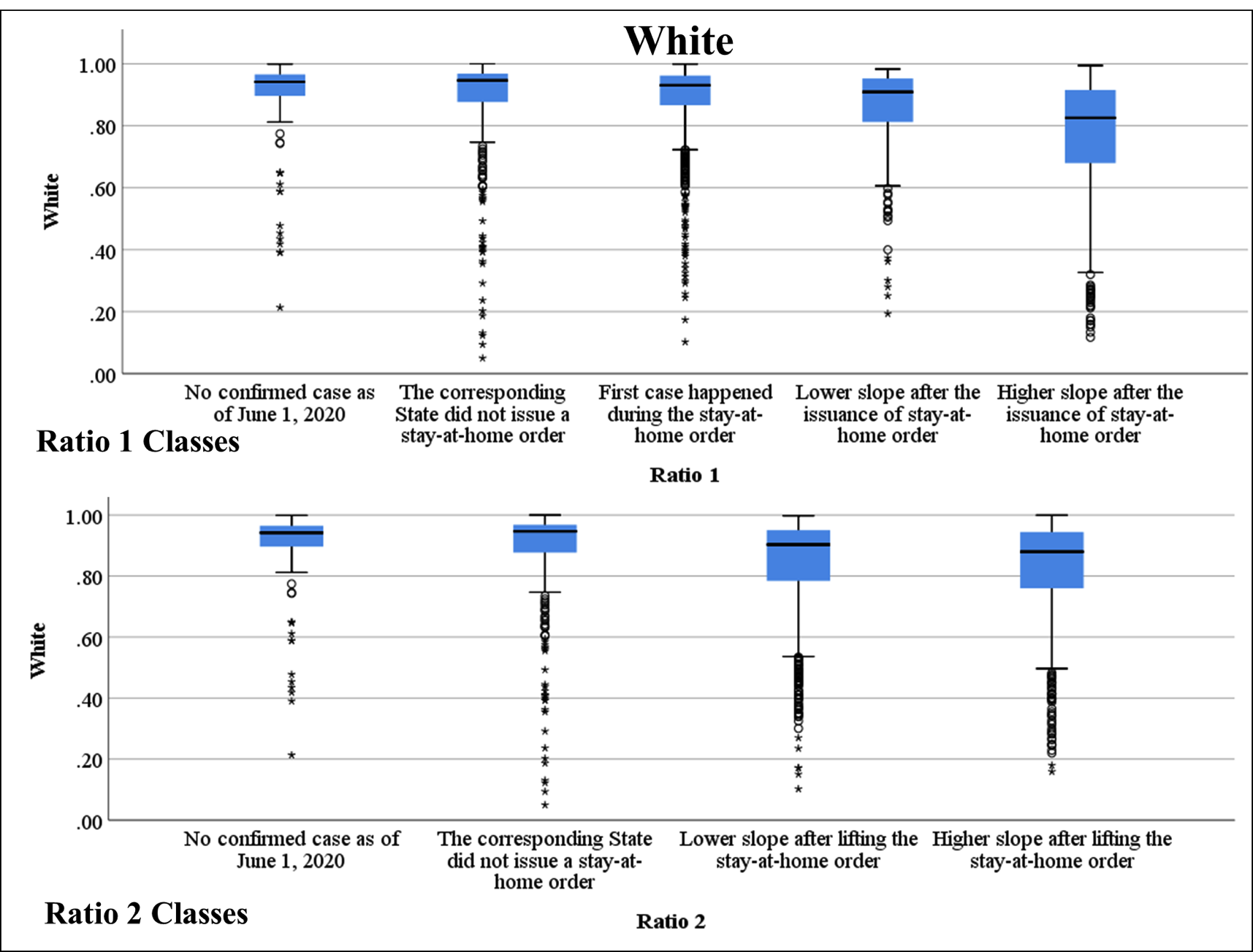


**Figure S17.** Distribution of White determinant among different groups defined based on Ratio 1 and Ratio 2. Ratio 1 compares the slope of the spread curve before and after the issuance of the Stay-at-home order while Ratio 2 compares the slopes before and after lifting the Order


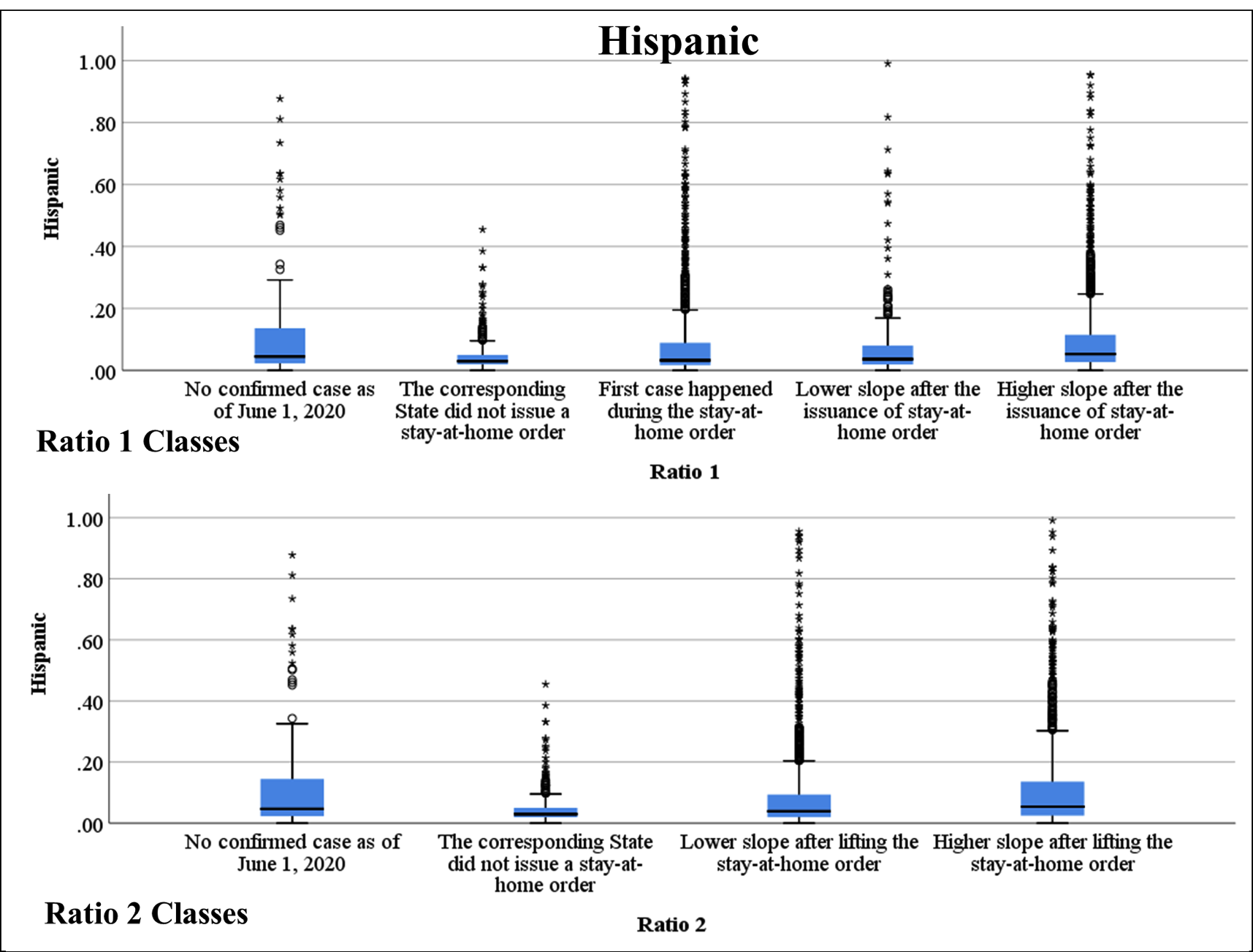


**Figure S18.** Distribution of Hispanic determinant among different groups defined based on Ratio 1 and Ratio 2. Ratio 1 compares the slope of the spread curve before and after the issuance of the Stay-at-home order while Ratio 2 compares the slopes before and after lifting the Order


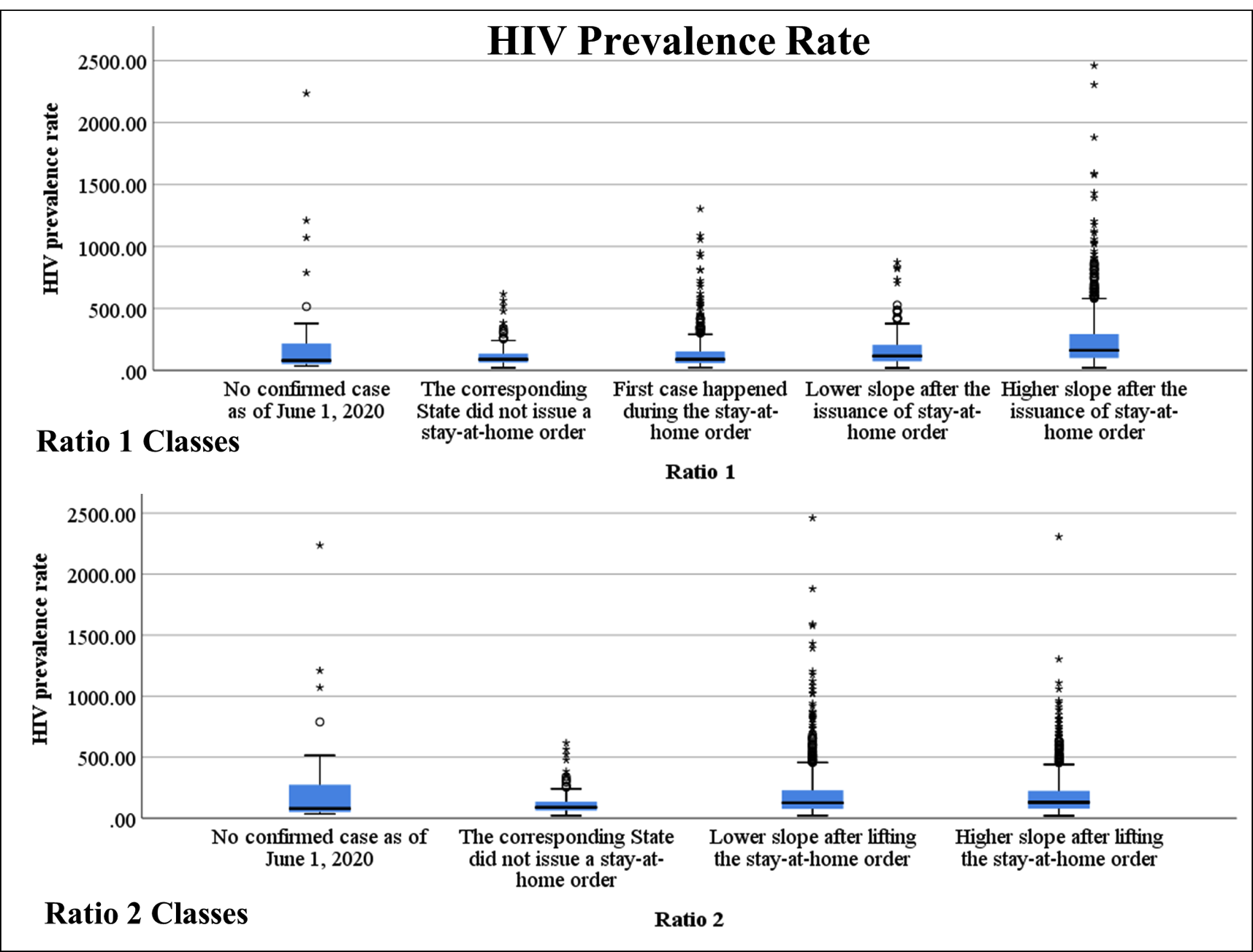


**Figure S19.** Distribution of HIV Prevalence determinant among different groups defined based on Ratio 1 and Ratio 2. Ratio 1 compares the slope of the spread curve before and after the issuance of the Stay-at-home order while Ratio 2 compares the slopes before and after lifting the Order


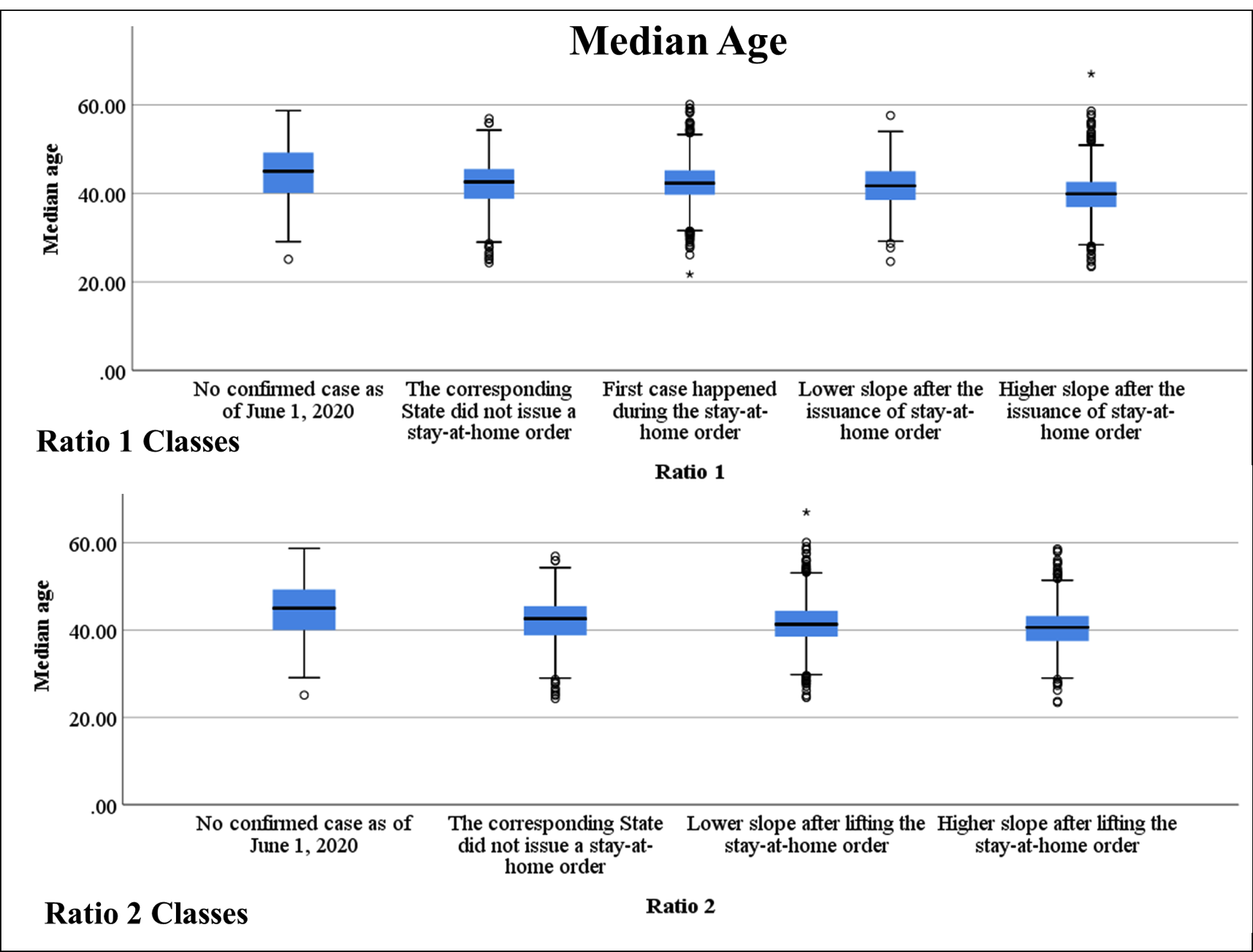


**Figure S20.** Distribution of Median Age determinant among different groups defined based on Ratio 1 and Ratio 2. Ratio 1 compares the slope of the spread curve before and after the issuance of the Stay-at-home order while Ratio 2 compares the slopes before and after lifting the Order


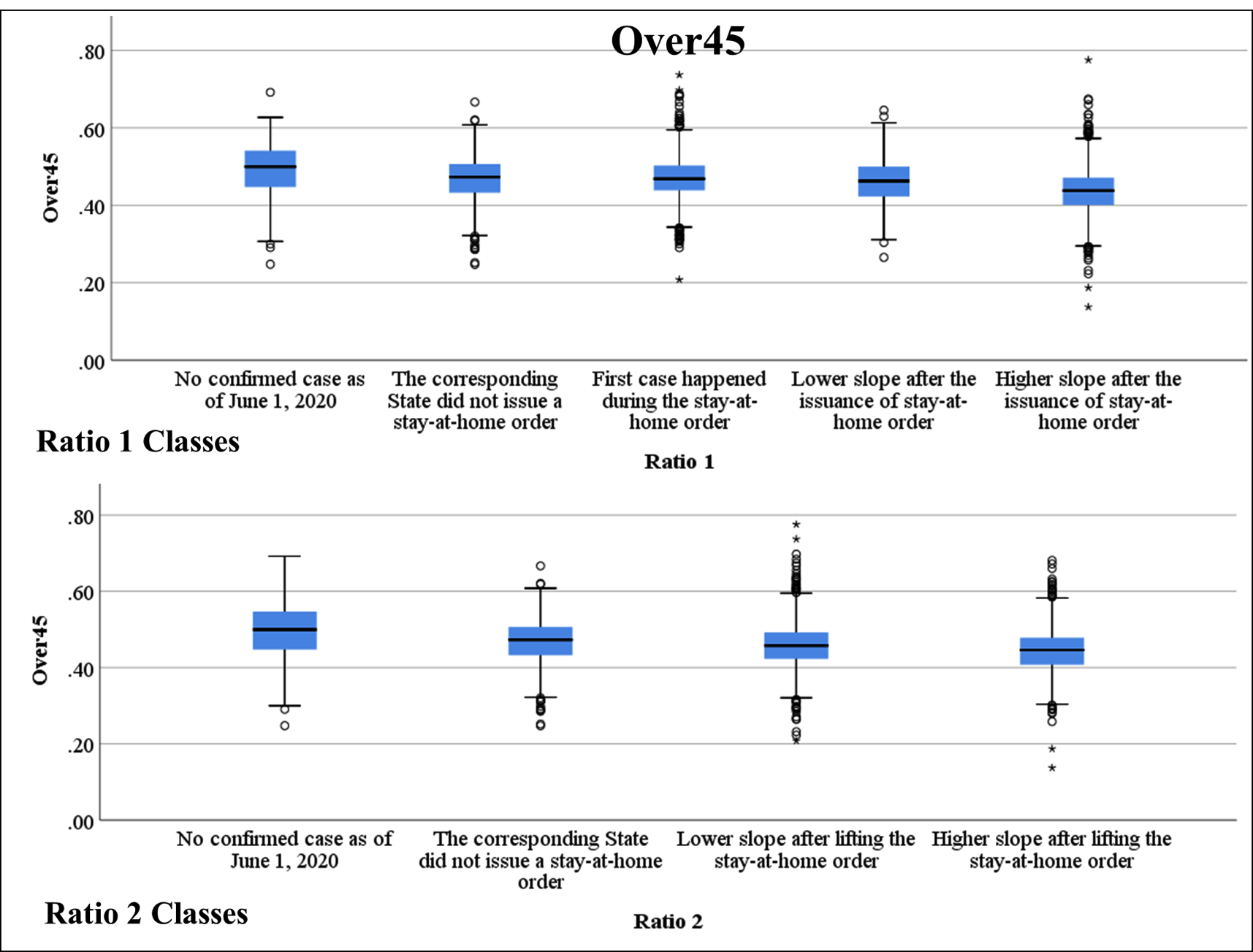


**Figure S21.** Distribution of Over 45 determinant among different groups defined based on Ratio 1 and Ratio 2. Ratio 1 compares the slope of the spread curve before and after the issuance of the Stay-at-home order while Ratio 2 compares the slopes before and after lifting the Order


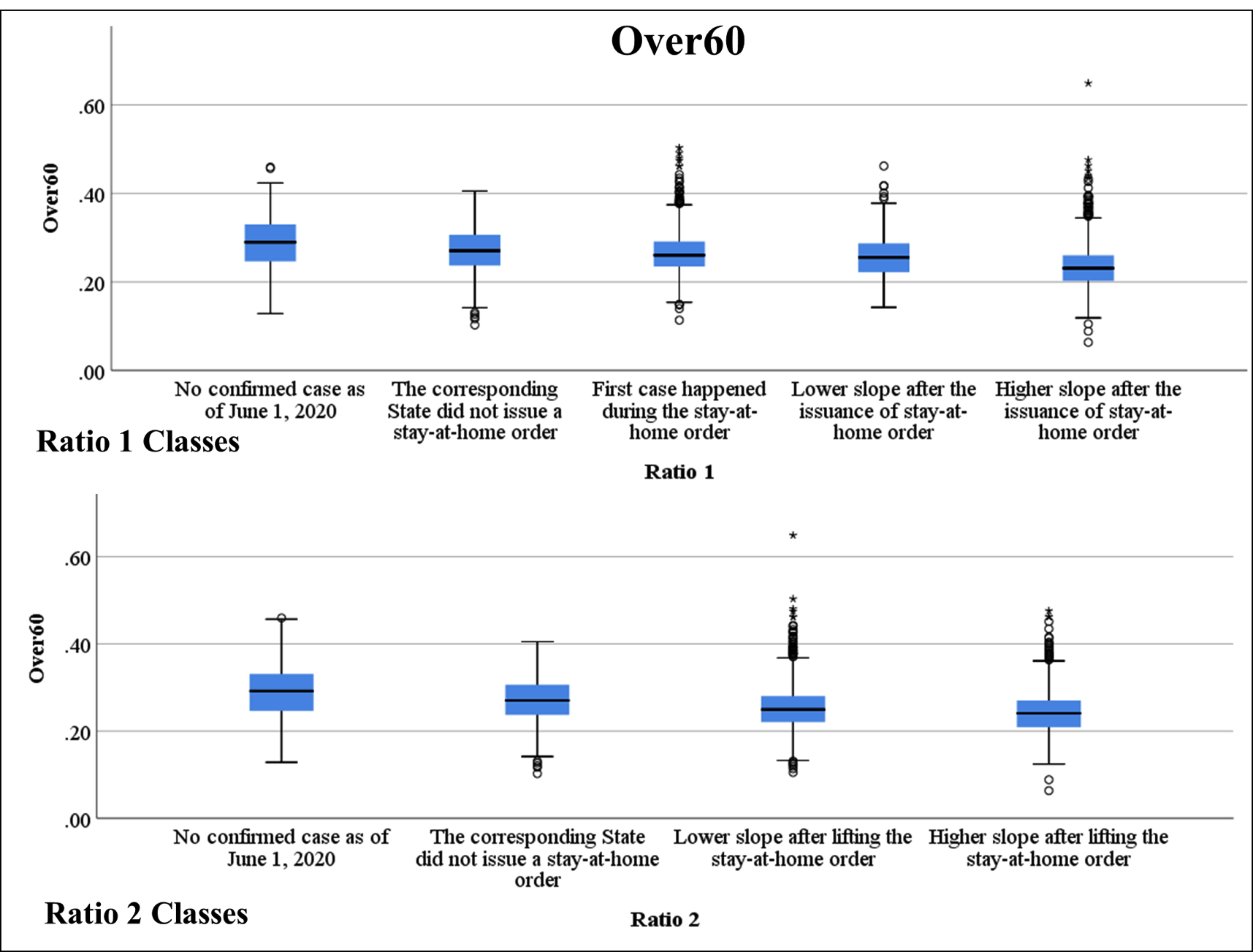


**Figure S22.** Distribution of Over 60 determinant among different groups defined based on Ratio 1 and Ratio 2. Ratio 1 compares the slope of the spread curve before and after the issuance of the Stay-at-home order while Ratio 2 compares the slopes before and after lifting the Order


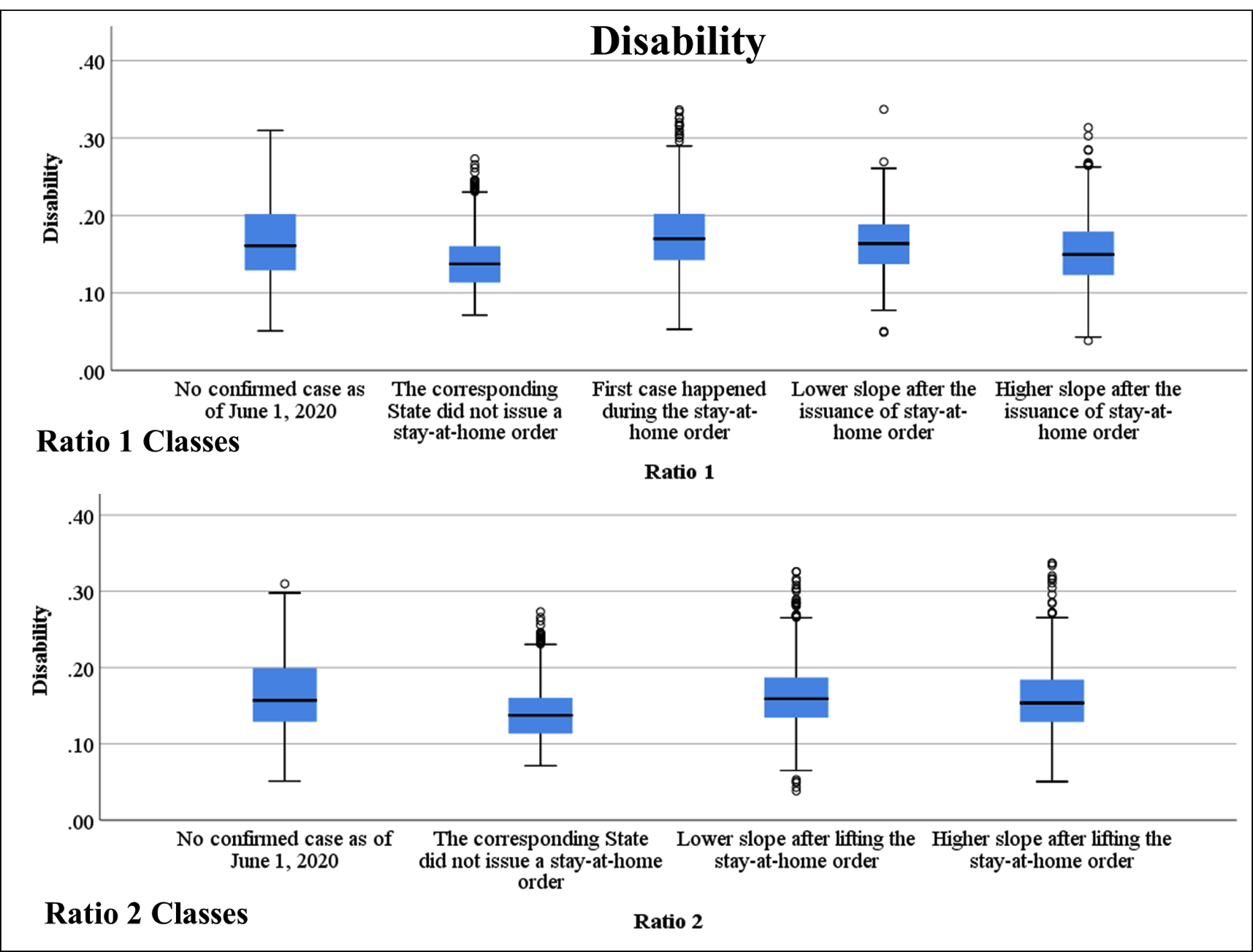


**Figure S23.** Distribution of Disability determinant among different groups defined based on Ratio 1 and Ratio 2. Ratio 1 compares the slope of the spread curve before and after the issuance of the Stay-at-home order while Ratio 2 compares the slopes before and after lifting the Order


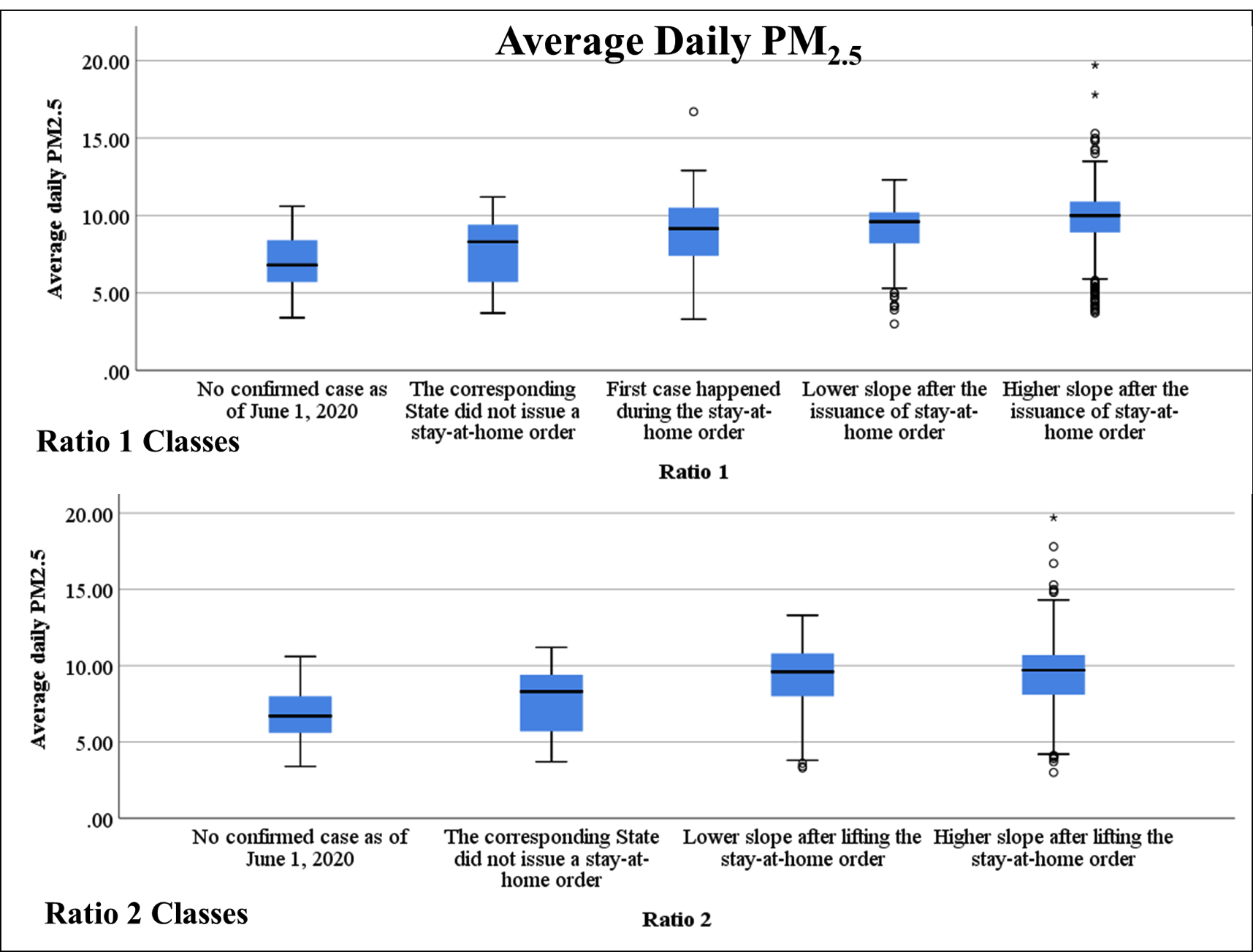


**Figure S24.** Distribution of Average Daily PM_2.5_ determinant among different groups defined based on Ratio 1 and Ratio 2. Ratio 1 compares the slope of the spread curve before and after the issuance of the Stay-at-home order while Ratio 2 compares the slopes before and after lifting the Order


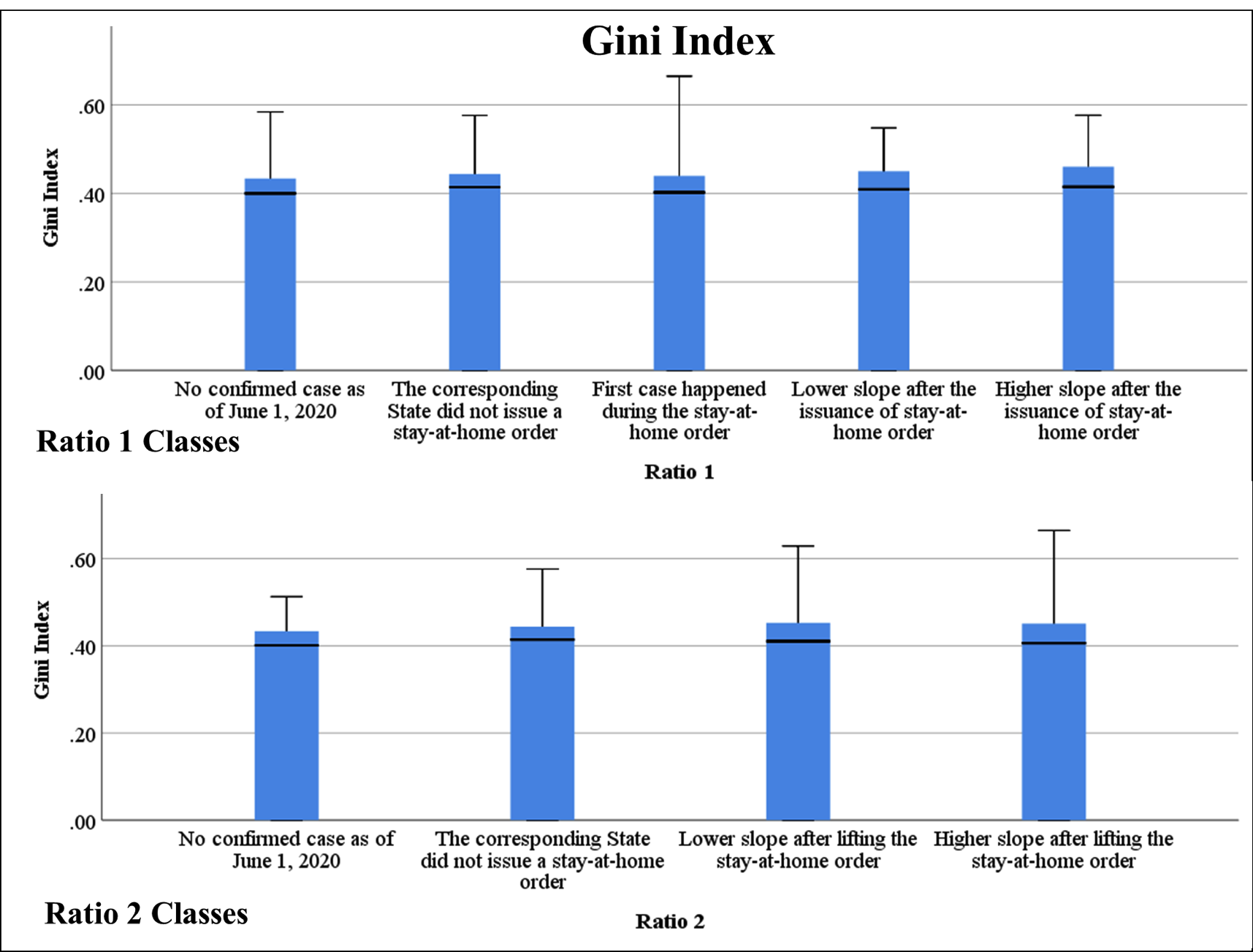


**Figure S25.** Distribution of Gini Index determinant among different groups defined based on Ratio 1 and Ratio 2. Ratio 1 compares the slope of the spread curve before and after the issuance of the Stay-at-home order while Ratio 2 compares the slopes before and after lifting the Order


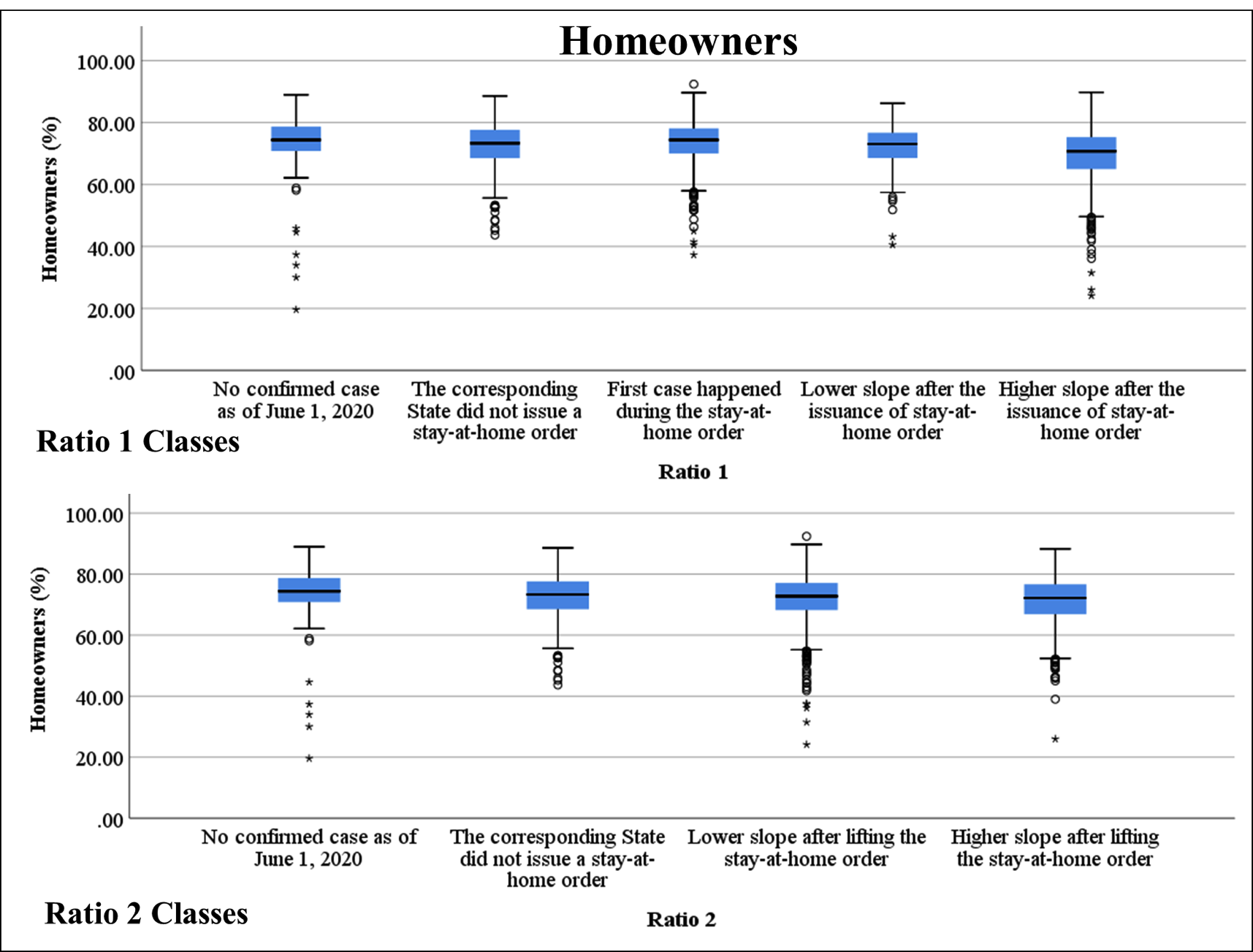


**Figure S26.** Distribution of Home Owners determinant among different groups defined based on Ratio 1 and Ratio 2. Ratio 1 compares the slope of the spread curve before and after the issuance of the Stay-at-home order while Ratio 2 compares the slopes before and after lifting the Order


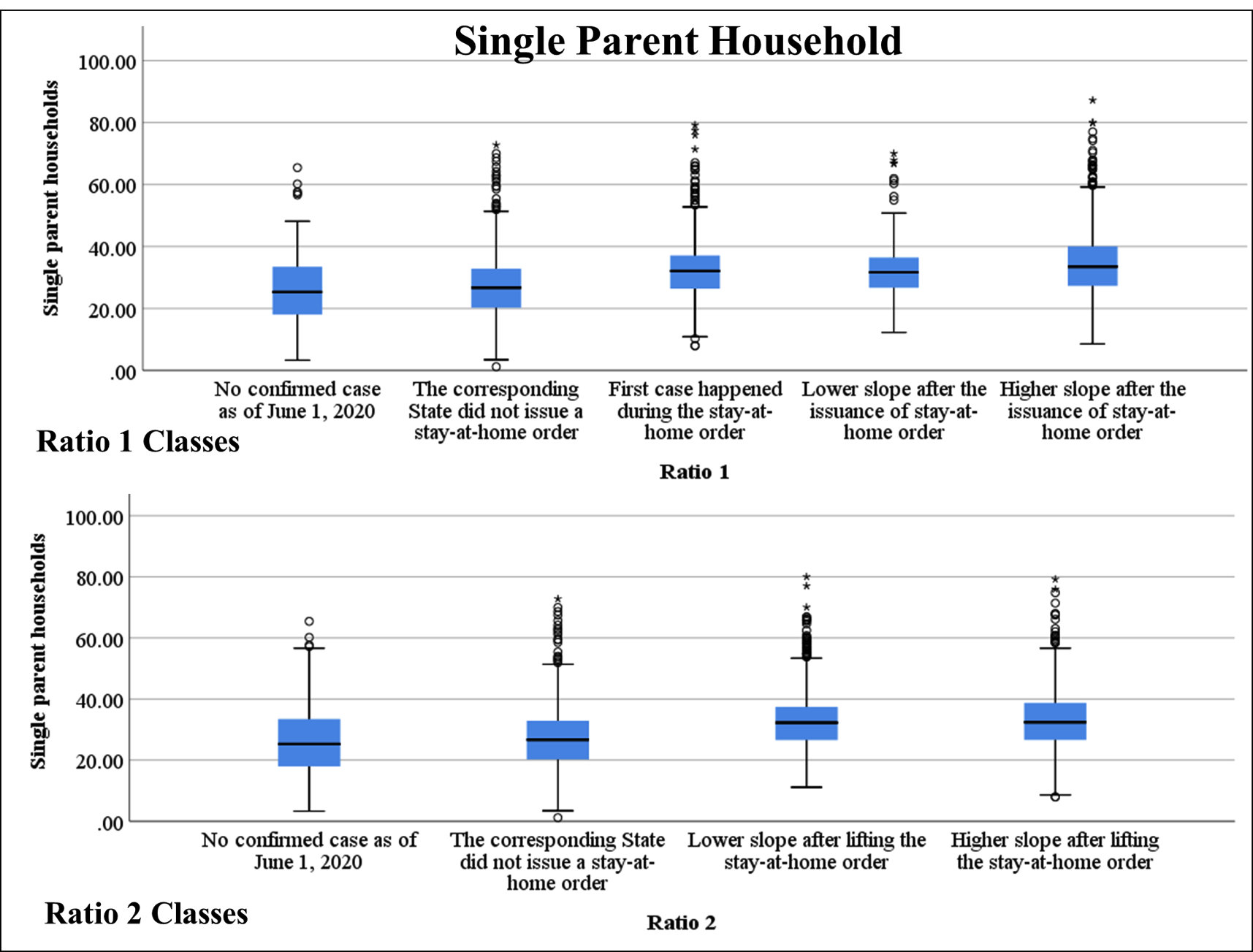


**Figure S27.** Distribution of Single Parent Households determinant among different groups defined based on Ratio 1 and Ratio 2. Ratio 1 compares the slope of the spread curve before and after the issuance of the Stay-at-home order while Ratio 2 compares the slopes before and after lifting the Order


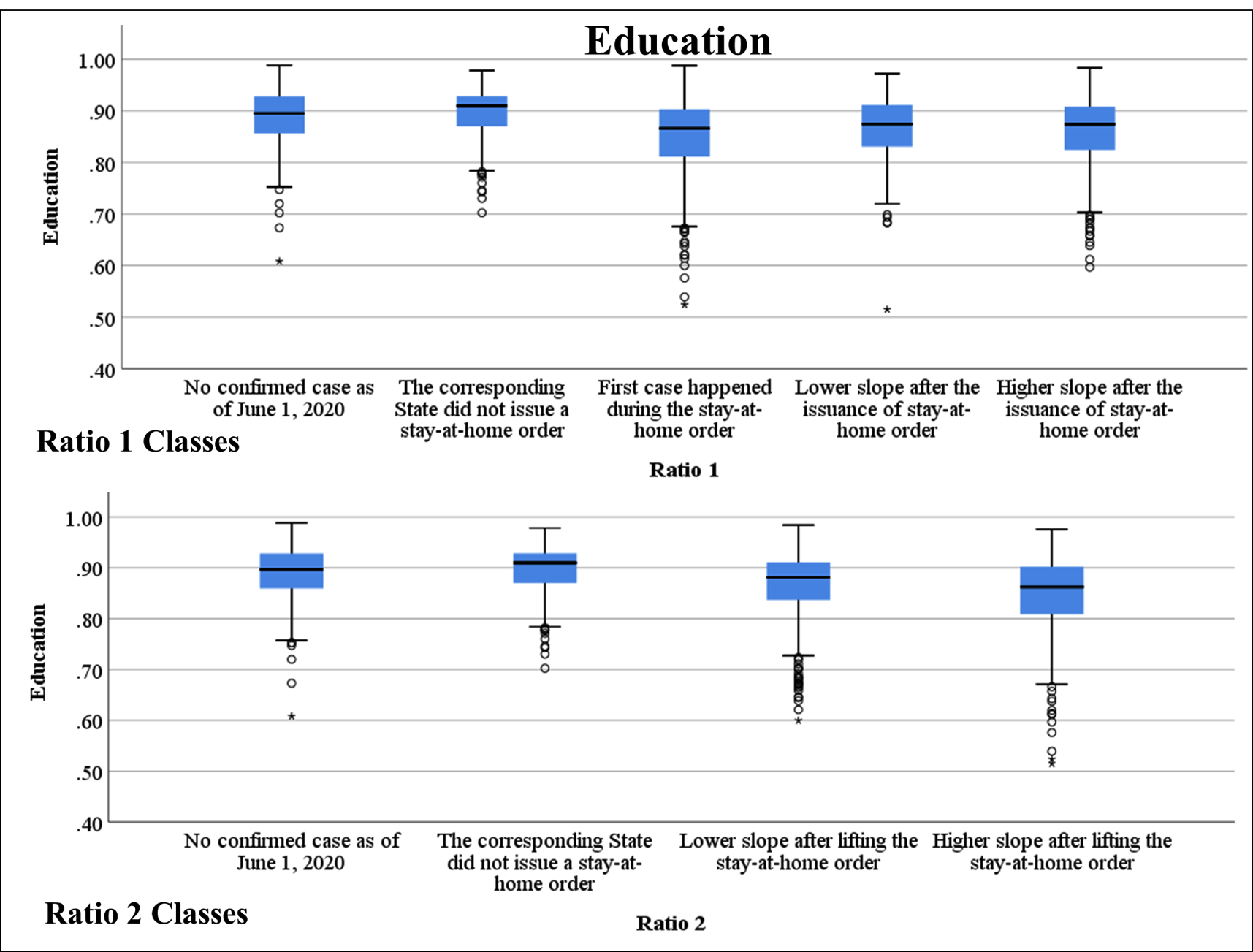


**Figure S28.** Distribution of Education determinant among different groups defined based on Ratio 1 and Ratio 2. Ratio 1 compares the slope of the spread curve before and after the issuance of the Stay-at-home order while Ratio 2 compares the slopes before and after lifting the Order


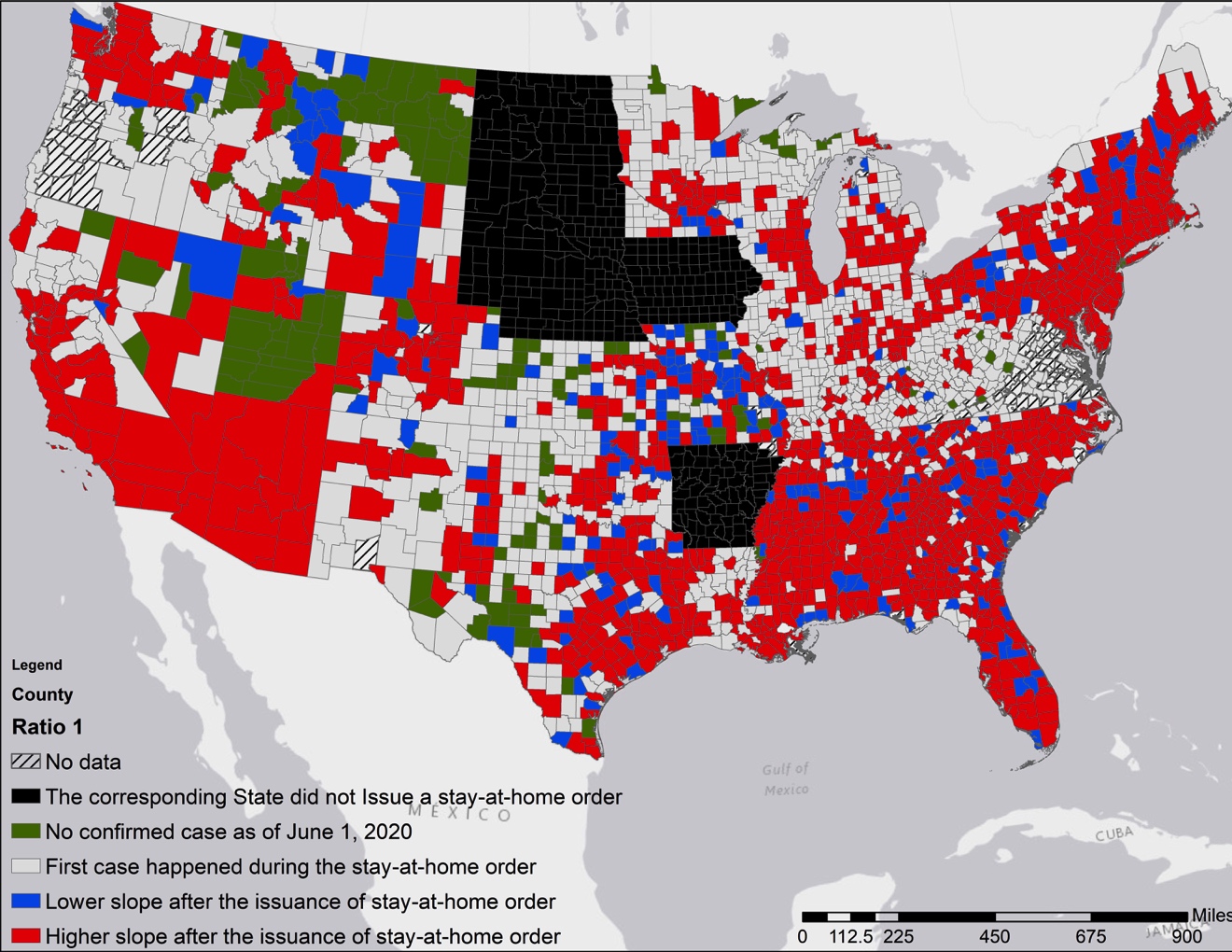


**Figure S29.** Geospatial distribution of counties wth regards to Ratio 1. Ratio 1 compares the slope of the spread curve before and after the issuance of the stay-home order


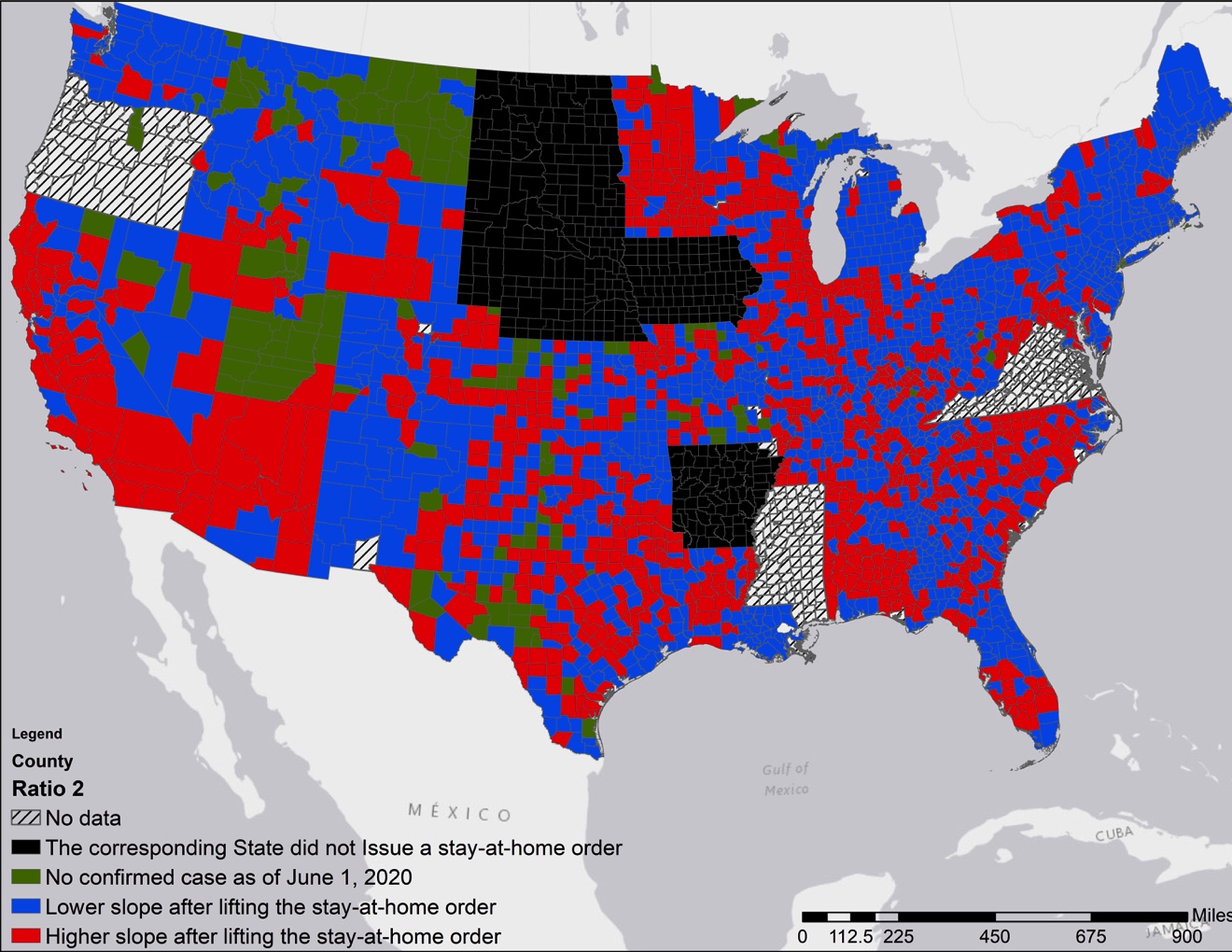


**Figure S30.** Geospatial distribution of counties wth regards to Ratio 2. Ratio 2 compares the slope of the spread curve before and after lifting of the Stay at Home Order.

**Table S1.** List of potential determinants sorted by absolute magnitude of their correlation (non-parametric Spearman's test and the corresponding Fisher Z-Transformed values) with the normalized COVID-19 Cases (as of June 1)

| **Variable** | **N** | **Correlation Coefficient (ρ)** | **Fisher Z-Transformed (****z′)** | **Sig. (2-tailed)** |
| --- | --- | --- | --- | --- |
| African-American | 3,087 | **0.491** | **0.537** | 2.1E-187 |
| White | 3,087 | **-0.440** | **-0.472** | 9.4E-148 |
| Population Density | 3,087 | **0.403** | **0.427** | 1.2E-120 |
| HIV Prevalence Rate | 2,152 | **0.403** | **0.427** | 1.1E-84 |
| Over60 | 3,087 | **-0.360** | **-0.377** | 1.5E-96 |
| Over45 | 3,087 | **-0.320** | **-0.332** | 1.6E-75 |
| Median Age | 3,087 | **-0.311** | **-0.322** | 1.7E-57 |
| Average Daily PM_2.5_ | 3,087 | **0.309** | **0.319** | 4.6E-69 |
| Single Parent Households | 3,085 | **0.247** | **0.252** | 5.2E-44 |
| Disability | 3,087 | **-0.210** | **-0.213** | 1.3E-33 |
| Homeowners | 3,087 | **-0.200** | **-0.203** | 1.6E-29 |
| Gini Index | 3,087 | **0.182** | **0.184** | 2.3E-24 |
| Hispanic | 3,087 | **0.168** | **0.170** | 4.9E-21 |
| Education | 3,087 | **-0.150** | **-0.151** | 7.2E-18 |
| Adults with Obesity | 3,087 | **0.126** | **0.127** | 2.5E-12 |
| Social Association Rate | 2,960 | **-0.120** | **-0.121** | 1.0E-11 |
| Fair or Poor Health | 3,087 | **0.116** | **0.117** | 8.7E-11 |
| Median Income | 3,087 | **0.101** | **0.101** | 1.7E-08 |
| Public Assistance | 3,087 | **0.096** | **0.096** | 9.2E-08 |
| Smokers | 3,087 | **0.087** | **0.087** | 1.1E-06 |
| Adults with Diabetes | 3,087 | **0.077** | **0.077** | 1.6E-05 |
| Unemployed | 3,087 | **0.041** | **0.041** | 2.3E-02 |
| Health Insurance | 3,087 | **-0.040** | **-0.040** | 2.4E-02 |
| Average Number of Mentally Unhealthy Days | 3,087 | **0.039** | **0.039** | 3.0E-02 |
| Average Number of Physically Unhealthy Days | 3,087 | 0.010 | 0.010 | 5.0E-01 |
| Food Insecure | 3,087 | 0.010 | 0.010 | 3.0E-01 |
| Physically Inactive | 3,087 | 0.010 | 0.010 | 2.8E-01 |
| Age-Adjusted Death Rate | 3,032 | 0.000 | 0.000 | 8.9E-01 |
| Excessive Drinking | 3,087 | 0.000 | 0.000 | 1.9E-01 |
| Life Expectancy | 3,019 | 0.000 | 0.000 | 2.7E-01 |
| Primary Care Physicians Ratio | 2,879 | 0.000 | 0.000 | 1.8E-01 |
| Teen Birth Rate | 2,944 | 0.000 | 0.000 | 4.1E-01 |

Bold values represent Spearman's correlation coefficient for variables with a significant difference at α=0.05

**Table S2.** County break down among different groups formed based on Ratio 1 and 2 values

| **Ratio 1 Classification** | **#** |
| --- | --- |
| No Confirmed Case as of June 1, 2020 | 145 |
| The Corresponding State Did not Issue a Stay-at-home order | 385 |
| First Case Happened During the Stay Home Period | 954 |
| Lower Slope after the Issuance of Stay-at-home order | 247 |
| Higher Slope after the Issuance of Stay-at-home order | 1253 |
| Total | 2984 |
|  | |
| **Ratio 2 Classification** | **#** |
| No Confirmed Case as of June 1, 2020 | 141 |
| The Corresponding State Did not Issue a Stay-at-home order | 385 |
| Lower Slope after Lifting the Stay-at-home order | 1289 |
| Higher Slope after Lifting the Stay-at-home order | 1023 |
| Total | 2838 |

**Table S3.** PDs with and without significant differences between higher and lower groups defined based on Ratio 1 and Ratio 2. Ratio 1 compares the slope of the spread curve before and after the issuance of the stay-home order while Ratio 2 compares the slopes before and after lifting the Order

| **Determinant** | **Ratio 1** | **Ratio 2** |
| --- | --- | --- |
| Median Age | Yes | Yes |
| Over45 | Yes | Yes |
| Over60 | Yes | Yes |
| Disability | Yes | Yes |
| White | Yes | Yes |
| Hispanic | Yes | Yes |
| Education | No | Yes |
| African-American | Yes | No |
| Population Density | Yes | No |
| Single Parent Households | Yes | No |
| Average Daily PM_2.5_ | Yes | No |
| HIV Prevalence Rate | Yes | No |
| Home Owner | Yes | No |
| Gini Index | No | No |
